# Pharmacometabolomics Study of Sulfamethoxazole and Trimethoprim in Kidney Transplant Recipients: Real-World Metabolism and Urinary Excretion

**DOI:** 10.3390/metabo15070473

**Published:** 2025-07-11

**Authors:** Marieke A. J. Hof, Hessel de Haan, Stepan Stepanovic, Stephan J. L. Bakker, Eelko Hak, Gérard Hopfgartner, Frank Klont, TransplantLines Investigators

**Affiliations:** 1 Department of Analytical Biochemistry, Groningen Research Institute of Pharmacy, University of Groningen, Antonius Deusinglaan 1, 9713 AV Groningen, The Netherlands; m.a.j.hof@rug.nl; 2 Unit of PharmacoTherapy, -Epidemiology & -Economics, Groningen Research Institute of Pharmacy, University of Groningen, Antonius Deusinglaan 1, 9713 AV Groningen, The Netherlandse.hak@rug.nl (E.H.); 3 Life Sciences Mass Spectrometry, Department of Inorganic and Analytical Chemistry, University of Geneva, Quai Ernest Ansermet 24, 1211 Genève, Switzerlandgerard.hofpgartner@unige.ch (G.H.); 4 Institute of Chemistry, Technology and Metallurgy, University of Belgrade, Njegoševa 12, 11000 Belgrade, Serbia; 5 Division of Nephrology, Department of Internal Medicine, University Medical Center Groningen, University of Groningen, Hanzeplein 1, 9713 GZ Groningen, The Netherlands; s.j.l.bakker@umcg.nl; 6 Department of Clinical Pharmacy and Pharmacology, University Medical Center Groningen, University of Groningen, Hanzeplein 1, 9713 GZ Groningen, The Netherlands; 7 Group of Authors on Behalf of the Transplant Lines Biobank and Cohort Study, University Medical Center Groningen, University of Groningen, Hanzeplein 1, 9713 GZ Groningen, The Netherlands; transplantlines@umcg.nl

**Keywords:** humans, metabolomics, pharmacometabolomics, mass spectrometry, cohort studies, xenobiotics, metabolism, SWATH

## Abstract

**Background/Objectives**: The increased use of antibiotics is raising concerns about environmental contamination and antibiotic resistance, exemplified by the case of cotrimoxazole, a widely prescribed combination of sulfamethoxazole and trimethoprim. After oral administration and absorption, both drugs are excreted in their parent and metabolized forms, which is a factor that is commonly considered in environmental studies. Many studies, however, rely on pharmacokinetic data from drug developers, who mostly investigate drug metabolism in healthy male volunteers rather than in actual patient populations. **Methods**: We investigated the real-world metabolism and urinary excretion of cotrimoxazole in an LC-SWATH/MS-based pharmacometabolomics study of 149 kidney transplant recipients who took part in the TransplantLines Biobank and Cohort Study (NCT0327284). **Results**: Our study confirmed (as “putatively characterized compound classes”) the presence of all the expected metabolites, and we (putatively) identified several previously unreported metabolites, including glucuronide conjugates of both drugs and two isoxazole ring-opened variants of sulfamethoxazole. The relative metabolite profiles furthermore indicated that the active drug trimethoprim accounted for 75% of the total signal intensity. For sulfamethoxazole, its acetylated metabolite was the main metabolite (59%), followed by the active parent drug (17%) and its glucuronide (7%). Alongside trimethoprim, these substances could serve as analytical targets for environmental cotrimoxazole monitoring, given their abundance (all three substances), activity (parent drug), and/or back-transformation potential (both conjugated metabolites). The isoxazole ring-opened variants (2–3%) may also warrant attention, considering their (presumed) absolute excreted quantities and potential pharmacological activity. **Conclusions**: This study underscores the value of pharmacometabolomics in elucidating real-world metabolite profiles, and it provides novel insights into cotrimoxazole metabolism and excretion, with implications for environmental and clinical monitoring.

## 1. Introduction

In recent years, the increased consumption of therapeutic drugs has raised concerns regarding the environmental consequences of pharmaceutical residues in the ecosystem [1]. These drugs mostly enter the sewage system upon excretion by humans via urine and feces and the subsequent flushing of these excrements down the toilet [2]. The toilet is, unfortunately, also frequently used as a waste bin for disposing of unused medicines, further exacerbating challenges in maintaining clean drinking water and in limiting the contamination of surface water with pharmaceutical residues [3].

The consequences of such contamination can affect the health and behavior of organisms living in this ecosystem and potentially pose risks to human health [4,5]. To illustrate this, medicines like painkillers can cause organ damage in fish, contraceptives can contribute to the feminization of male fish, antidepressants can alter fish behavior, and antibiotics can increase bacterial resistance [5,6,7,8]. This last group of medicines in particular is raising increasing concern, with antibiotic resistance genes having been discovered in microorganisms found in groundwater, surface water, sewage water, wastewater treatment plants, and even drinking water [9].

Within the class of antibiotics, sulfamethoxazole and trimethoprim, often prescribed in combination under the name cotrimoxazole, are notable examples of therapeutic drugs which are currently being monitored closely [10]. For example, in the case of sulfamethoxazole, its levels detected in environmental samples occasionally exceed the so-called predicted no-effect concentration (PNEC) threshold value, which resides in the low microgram per Liter range, indicating a risk to aquatic ecosystems [11,12]. Moreover, high picogram to low nanogram per Liter levels have also been found in water intended for human consumption, which suggests that this drug’s removal by wastewater treatment plants is substantial but incomplete [13].

An important aspect of current environmental drug monitoring practices and the assessment of drugs’ ecotoxicological hazard potential is the recognition that many drugs are partially excreted as metabolites rather than in their originally administered form [14,15,16,17,18]. This understanding has led to the inclusion of so-called excretion fractions in many environmental studies, often relying on pharmacokinetic data reported by pharmaceutical companies [2,17,18,19]. It is, however, often overlooked that the corresponding metabolism and excretion data are generally obtained through (highly complex and costly) mass balance studies, typically including around five healthy male volunteers receiving a single dose of the investigational drug [20]. Consequently, the generalizability of these data to real-world drug users may be limited, potentially leading to under- or overestimations of the real risks associated with these drugs for the aquatic milieu. Larger-scale studies of drug metabolism and excretion in actual patient populations are thus needed, for example, utilizing a liquid chromatography and high-resolution mass spectrometry (HRMS)-based pharmacometabolomics (PMx) approach [21].

In the case of cotrimoxazole, immunosuppressed humans represent an interesting study domain as these individuals receive this drug combination prophylactically for a longer period [22]. This population encompasses individuals with a primary (inherited or congenital) immunodeficiency disorder, such as common variable immune deficiency (CVID), as well as those with secondary (acquired) immunodeficiency disorders, which may result from diseases like HIV/AIDS or are as a result of immunosuppressive drug therapies [23]. The latter, drug-induced immunosuppression, is particularly prevalent in kidney transplantation, where transplant recipients are routinely prescribed cotrimoxazole prophylaxis to prevent infections caused by *Pneumocystis jirovecii* (PCP) [22,24].

In this study, we elucidated the real-world metabolism of cotrimoxazole utilizing a pharmacometabolomics approach applied to large numbers of 24 h urine samples from kidney transplant recipients contributing to the TransplantLines Biobank & Cohort Study [25]. The resulting data were compared with existing information on the metabolism of trimethoprim and sulfamethoxazole to confirm the presence of previously identified metabolites and potentially uncover previously unreported ones. The latter are of particular interest since the drugs’ kinetics, as established in healthy volunteers, may not be generalizable [26] to the prolonged drug use in the selected study population of organ transplant recipients. Additionally, informed suggestions of target compounds to be included in environmental monitoring analyses are provided.

## 2. Materials and Methods

### 2.1. Clinical and Pharmacometabolomics Data

This study utilized existing 24 h urine pharmacometabolomics data of 163 kidney transplant recipients for whom samples had been taken at 3, 12, and 24 months after transplantation. PMx analyses were performed in an untargeted, data-independent acquisition (DIA) mode using reversed-phase liquid chromatography coupled with time-of-flight (TOF) mass spectrometry conducted in the ‘SWATH’ acquisition mode, first described by Hopfgartner et al. [27] and Gillet et al. [28] in 2012. A complete and detailed description of the corresponding PMx analyses has been given elsewhere [21], and the PMx data have been deposited in an open-access data repository, which can be found at https://doi.org/10.26037/yareta:64ruex2sxff5nenyfyexurzs3m (as sub-study 5). The underlying clinical study, the TransplantLines Biobank and Cohort Study (NCT identifier ‘NCT03272841’) [25], was approved by the Institutional Review Board of the University Medical Center Groningen (UMCG; decision METc 2014/077 on 25 August 2014), adhered to the UMCG Biobank Regulation, the Declaration of Helsinki, and the Declaration of Istanbul, and collected informed consent from all the participants. Clinical data were retrieved from medical records (e.g., age, sex, transplantation details) and through measurements during and shortly after the visits (e.g., of the body mass index, estimated glomerular filtration rate (eGFR; CKD-EPI creatinine–cystatin equation 2021), serum albumin, and serum alanine aminotransferase). The drug exposure was based on the self-reported use, with the exception of sulfamethoxazole and trimethoprim, for which the determination of the drug exposure relied on their presence in the urinary PMx data (see Figure S1).

### 2.2. Feature Selection

SCIEX MarkerView software (version 1.3.1) was used for extracting and aligning two-dimensional features (i.e., mass-to-charge ratio (*m*/*z*), retention time) from the raw data, with the settings presented in Table S1. Statistically significant features (Student’s *t*-test: α = 0.05, with Bonferroni correction) were subsequently extracted using the PMx-based sulfamethoxazole exposure as the grouping variable. Next, the features were excluded in cases of low abundance (i.e., a median signal intensity below 1.0% of the median intensity observed for trimethoprim), that displayed the wrong association direction (i.e., negative log fold change), or that did not correspond to a precursor ion (i.e., isotopes, in-source fragments, adducts).

### 2.3. Metabolite Identification

To account for the difficulties in resolving closely eluting peaks using automated feature detection tools, exposure-positive samples were assessed manually with SCIEX PeakView (version 2.2.0.11391), taking into account the retention times and *m*/*z* values of the selected features. Specifically, the corresponding extracted ion chromatograms and underlying SWATH/MS fragment spectra were screened for peaks that could reflect sulfamethoxazole or trimethoprim metabolites. This screening took into account several factors, including these compounds’ chemical properties (e.g., their molecular weight, molecular formula, retention times, and known fragment spectra and the fragmentation spectra of chemical reference standards (when available) measured separately), existing information on their metabolism (e.g., acetylation, dealkylation, glucuronidation, oxygenation), and the principles of drug metabolism in humans and the analytical workflow employed (e.g., reversed-phase liquid chromatography, positive electrospray ionization, collision-induced dissociation). This manual identification was performed independently by two researchers. For all (putative) sulfamethoxazole and trimethoprim metabolites, the signal intensities were subsequently extracted manually from exposure-positive samples at three months after transplantation, which was the most relevant timepoint due to cotrimoxazole prophylaxis typically being prescribed during the first six months after transplantation. This extraction was performed using SCIEX MultiQuant software (version 2.1) with a 2.0-point Gaussian smoothing width and a ±5 mDa mass extraction window. Next, for both drugs, the resulting data were used to calculate relative metabolite profiles by dividing the signal intensity of each individual substance by the sum of the signal intensities of all the substances found per drug user. Finally, representative samples were reanalyzed for complementary fragment spectra using TOF mass spectrometry in the product ion scan mode with a collision energy ranging from 10 to 70 eV. The selected samples originated from an aliquot set that is commonly used for confirmatory experiments.

## 3. Results

### 3.1. Characteristics of Study Participants

The kidney transplant recipients included in this study were mostly male with a median age of 58 years (see Table 1). All but three participants were using a prophylactic agent for PCP three months post-transplantation. Among the participants using a prophylactic agent, 1 used pentamidine (one monthly dose of 300 mg), 7 used atovaquone monotherapy (one daily dose of 1500 mg), 2 used a combination of atovaquone (one daily dose of 1500 mg) and trimethoprim (one daily dose of 100 mg), and 149 used cotrimoxazole. With respect to the latter group, most received a daily dose of 400/80 mg. Four individuals, however, received this dose once every two days, one received double this dose, and one participant reported usage without specifying the dose. Lastly, at 12 and 24 months post-transplantation, most kidney transplant recipients were no longer using these drugs, although the molecular evidence of sulfamethoxazole and trimethoprim exposure suggests the possible underreporting of trimethoprim use.

### 3.2. Feature Selection

Starting with 103,681 features, 1033 were significantly associated with PMx-verified sulfamethoxazole exposure (exposed vs. nonexposed samples, from any timepoint), which was used as a proxy for cotrimoxazole use given that this drug is only used in combination with trimethoprim while the latter is also used individually. After removing isotopes, adducts, in-source fragments, and low-abundance features as well as features showing lower mean values in the exposed group compared to the nonexposed group, 23 features remained (Table 2). Thirteen of these features, including the one corresponding to sulfamethoxazole, had even *m*/*z* values, while ten, including the one corresponding to trimethoprim, had odd *m*/*z* values.

### 3.3. Metabolite Identification

The manual assessment of the exposure-positive samples using the *m*/*z* values and retention times of the 23 prioritized features revealed 13 distinct signals associated with sulfamethoxazole exposure (see Figures S2–S9 and Table 3 and Table S2), all having even *m*/*z* values. Twelve distinct signals were further found to be associated with trimethoprim exposure (see Figures S10–S15 and Table 4 and Table S3), all having odd *m*/*z* values.

**Figure 1:**
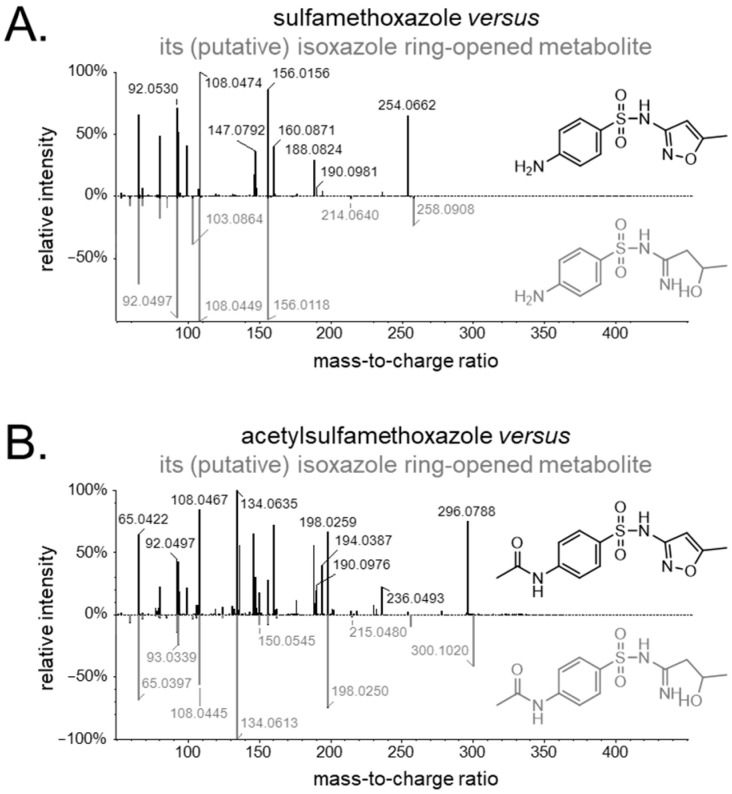
Exemplary fragment spectra (product ion scan mode, collision energy of 40 ± 30 eV) and structural formulas of (**A**) sulfamethoxazole and (**B**) acetylsulfamethoxazole as well as their (putative) isoxazole ring-opened variants, as observed in the urine of a human cotrimoxazole user.

In the case of sulfamethoxazole, the signals for sulfamethoxazole and acetylsulfamethoxazole were the highest, corresponding to 17% and 59%, respectively, of the total intensity observed for the 13 identified substances associated with sulfamethoxazole use. Most of the other substances featured median metabolite abundances of up to 1%, with the exception of a glucuronidated version of sulfamethoxazole (7%), an oxygenated version of acetylsulfamethoxazole (5%), and the isoxazole ring-opened variants of both substances (2–3%). With respect to the latter (see Figure 1), this presumed biotransformation step was prompted by existing information on the metabolism of leflunomide, an isoxazole derivative used in rheumatoid arthritis [29]. This immunosuppressive prodrug is primarily metabolized to its active isoxazole ring-opened metabolite with a +4.031 Da mass shift, thus matching the mass shifts we observed for sulfamethoxazole and acetylsulfamethoxazole, which, too, are isoxazole derivatives. Lastly, quantum chemistry-based simulations using Koopman and Grimme’s method [30] (see Data S1) supported the plausibility of the corresponding biotransformation reaction for both substances. The simulations evaluated the imine, ketone, and enol forms of the proposed substance, finding the imine both to be the most thermodynamically stable and to best match the experimental MS/MS spectra across the collision energies, notably reproducing the characteristic *m*/*z* 103 fragment shown in Figure 1A.

In the case of trimethoprim, the administered (and active) substance itself accounted for 75% of the total intensity observed for the 12 identified substances associated with trimethoprim use. Most of the other substances featured median metabolite abundances of between 1% and 6%, with the exception of two trimethoprim glucuronides and a sulfate conjugate of demethyl trimethoprim, for which the abundances were 0.5% or lower.

**Figure 2:**
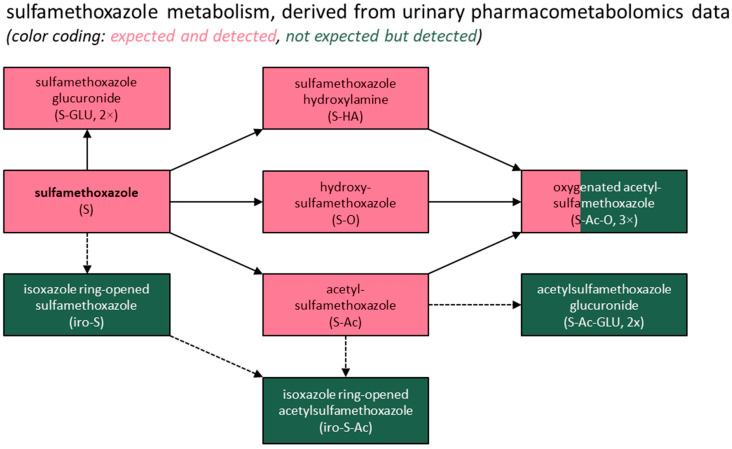
Summary of the study findings for sulfamethoxazole. A more detailed overview of the putative human metabolism of sulfamethoxazole based on this study’s findings, including structural formulas, is included in Supplementary Figure S16.

**Figure 3:**
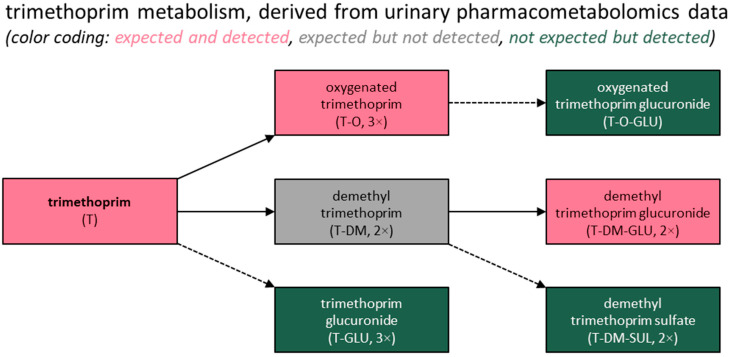
Summary of the study findings for trimethoprim. A more detailed overview of the putative human metabolism of trimethoprim based on this study’s findings, including structural formulas, is included in Supplementary Figure S17.

Lastly, all the metabolites expected to be present based on a previous report [31] were detected in this study, except for two demethylated forms of trimethoprim. However, glucuronide conjugates of these two metabolites were found, consistent with the previous report [31] stating that both are “excreted partly as such, but mainly conjugated with glucuronic acid.” Additionally, several previously unreported metabolites were identified for both drugs, most of which were phase II conjugates, with the exception of the isoxazole ring-opened metabolites of sulfamethoxazole. These findings are summarized in Figure 2 and Figure 3.

## 4. Discussion

Our pharmacometabolomics study confirmed the presence of all the (logically) expected [31] metabolites of trimethoprim and sulfamethoxazole in human urine samples from around 150 real-world drug users within a transplantation setting. In addition, we found various unreported metabolites, which mostly comprised phase II metabolites (i.e., sulfate and glucuronide conjugates). For sulfamethoxazole, we also found four unreported phase I metabolites, namely two oxygenated variants of acetylsulfamethoxazole as well as isoxazole ring-opened variants of both sulfamethoxazole and its main metabolite, acetylsulfamethoxazole. The latter type of conversion is rather uncommon and is not typically mentioned in lists of common drug biotransformation reactions [32]. However, it has previously been observed for another, structurally rather dissimilar isoxazole derivative, leflunomide, where it represented the activation reaction of this antirheumatic prodrug, which reversibly inhibits the mitochondrial enzyme dihydroorotate dehydrogenase, thus interfering with de novo pyrimidine nucleotide biosynthesis [29]. Moreover, isoxazole ring opening has been described for sulfamethoxazole in (bio)degradation experiments, although the corresponding (intermediate) products were of different molecular masses, indicating that they were distinct substances [33,34,35].

When assessing the relative metabolite abundances, trimethoprim itself produced by far the highest-intensity signals, accounting for approximately 75% of the total signal intensities observed for all the trimethoprim-associated signals. Due to the incomparable (relative) quantification principles of the techniques utilized, this relative contribution cannot be compared directly with previously obtained mass balance data [31] showing a 77.5% recovery of the unaltered drug in urine. Given that the latter data type is typically used for calculating excretion fractions in environmental research, the relative contribution of trimethoprim that we found appears consistent with the excretion fractions reported in recent environmental studies [2,36]. Additionally, it may reasonably be concluded that the active pharmaceutical ingredient trimethoprim represents a sensitive, relevant, and logical target compound for qualifying and quantifying humans’ exposure to this drug through the analysis of human excreta. Furthermore, when taking into account that trimethoprim is considered to be chemically stable in various environmental samples [37,38], trimethoprim itself would also be an appropriate target compound for the detection of contamination in environmental contexts.

In the case of sulfamethoxazole, the substances expected to be found in urine [31] accounted for approximately 90% of the total signal intensities observed for all the sulfamethoxazole-associated signals, with sulfamethoxazole (16.5%) and its acetylated metabolite (59.2%) being the most abundant substances. A direct comparison with previously obtained quantitative data is also not possible for this drug, but previous mass balance studies [31] did show rather comparable urinary fractions of 20% and 60–65%, respectively, of which the former percentage has also been observed in recent environmental studies [2,36] as an excretion fraction for sulfamethoxazole. Both compounds thus seem logical target compounds to use to qualify and quantify the human exposure to this drug upon the analysis of human excreta. Furthermore, acetylsulfamethoxazole seems to be a rather sensitive compound, as supported by previous studies, in spite of some suggesting this metabolite’s potential (partial) back-transformation into unconjugated sulfamethoxazole [39,40,41]. Admittedly, acetylsulfamethoxazole’s reduced biological activity/toxicity compared to sulfamethoxazole may limit the relevance of measuring it [12,42,43]. Nonetheless, it could be a valuable target considering its back-transformation potential and the varying sulfamethoxazole removal rates previously reported for different wastewater treatment plants [12,39].

Regarding the other detected metabolites, most represented the glucuronide or sulfate conjugates of a limited set of known substances. A potential assessment of their ecotoxicity could be limited to only a few substances, notably under the assumption that phase II metabolites back-transform into their respective unconjugated forms. The latter assumption should, however, be applied cautiously in the case of sulfamethoxazole, especially since its N-glucuronidated form (along with its N-acetylated form) has been detected even after wastewater treatment processing [41]. Consequently, including this substance in testing panels has been proposed to provide a more accurate estimation of the sulfamethoxazole removal rates, particularly given that back-transformation may distort these rates when occurring disproportionally before and after treatment [12].

Two metabolites requiring particular attention are the isoxazole ring-opened variants of sulfamethoxazole and acetylsulfamethoxazole. Admittedly, these unreported metabolites accounted for only 1.6% and 3.0% of the total signal intensities observed for all the sulfamethoxazole-associated signals, respectively. This drug, however, is frequently prescribed in gram amounts, so the absolute amount excreted for a metabolite with a relative contribution of around 2–3% would still exceed the defined daily dosage of a drug like fluoxetine, which is also a substance of environmental concern. Given such an amount, these metabolites could possibly be studied within a clinical context as well, to assess their potential pharmacological activity and toxicity in humans.

Lastly, a notable strength of this study is that it provides urinary excretion data from a large number of real-world cotrimoxazole users, thus capturing considerable variability due to anthropometric, demographic, exposure, genetic, and (patho)physiological factors (although not correcting for these factors). However, we acknowledge that our study only includes individuals from a single, confined geographical area and from a transplantation setting, while cotrimoxazole is used globally, including chronically by various other groups of immunocompromised individuals. Additionally, we applied untargeted analytical techniques (in a non-regulatory environment) to biobanked samples, which are inherently subject to multiple sources of (pre)analytical bias, including the timing of cotrimoxazole intake relative to the 24 h urine collection window and storage conditions. Furthermore, the technique employed only allowed us to obtain a relative quantitative estimate, unlike, for example, properly validated targeted approaches and radioactivity-based detection techniques (as used in radiolabeled mass balance studies), which limits the interpretation of the results and calls for further studies to be carried out. Despite most of the reported substances being ‘level 3’ identifications, or belonging to ‘putatively characterized compound classes’ (focusing on their accurate mass and spectral similarity to known compounds) [44], according to the Metabolomics Standards Initiative [45], these were solely observed in samples from individuals exposed to cotrimoxazole. This supports their designation as potential trimethoprim or sulfamethoxazole metabolites, or at least as substances associated with cotrimoxazole use. Nonetheless, we acknowledge the importance of conducting further confirmatory studies, notably involving (bio)synthesized substances and complementary analytical techniques. Lastly, we acknowledge that the metabolite patterns detected in the biobanked samples may not fully reflect those at the time of excretion into the urine bladder (or even at the time of collection) or entry into the ecosystem, underscoring the need to replicate this study, for example, by utilizing freshly collected human samples and/or wastewater samples. By working with biobanked 24 h urine collection samples, however, it was unlikely that highly unstable or labile metabolites would have been detected, thus suggesting that the metabolites reported may potentially also be detectable in environmental matrices, such as wastewater, and could be targeted in future studies.

## 5. Conclusions

This pharmacometabolomics study identified (as “putatively characterized compound classes”) all the expected and multiple previously unreported metabolites of trimethoprim and sulfamethoxazole in 24 h urine samples from around 150 chronic cotrimoxazole users. Our findings also suggest that trimethoprim, sulfamethoxazole, and acetylsulfamethoxazole (and potentially also sulfamethoxazole glucuronide) are logical compounds to target analytically, both in terms of their analytical sensitivity and biological relevance. Moreover, while direct quantitative comparisons with mass balance data were not feasible, the observed metabolite profiles broadly align with the existing mass balance data reported for trimethoprim and sulfamethoxazole and thus with the excretion fractions recently used in environmental studies. Furthermore, particular attention should be given to the newly described isoxazole ring-opened metabolites of sulfamethoxazole and acetylsulfamethoxazole, which were detected in all the samples. While their relative abundance is low, their absolute quantities may still be substantial, which would be especially important if these metabolites have high intrinsic activity. These findings thus underscore the importance of further studying these metabolites, particularly in the context of their pharmacological effects in humans and possibly also regarding their potential environmental impact.

## Figures and Tables

**Table 1:** Characteristics of the 163 kidney transplant recipients included in this study.

Variable	At 3 Months	At 12 Months	At 24 Months
Age (years), median (IQR)	58 (48–65)		
Female (%)	34		
Living kidney donation (%)	70		
BMI (kg/m^2^), median (IQR)	27 (24–29)	27 (24–30)	27 (24–30)
Serum albumin (g/L), median (IQR)	44 (42–46)	44 (42–46)	44 (42–46)
Serum ALT (U/L), median (IQR)	20 (15–26)	20 (16–26)	20 (16–26)
eGFR (mL/min/1.73 m^2^), median (IQR)	48 (38–58)	51 (40–59)	53 (41–63)
*Self-reported PCP prophylactic agent use*			
Cotrimoxazole (%)	91	7	3
Trimethoprim (%)	1 ^a^	1	1
Atovaquone (%)	6 ^a^	0	0
Pentamidine (%)	1	0	0
*Analytical evidence of (presumed) drug exposure*			
Sulfamethoxazole (%)	93	6	3
Trimethoprim (%)	96	14	6
*Self-reported immunosuppressant use*			
Cyclosporine (%)	2	6	6
Tacrolimus (%)	98	93	93
Azathioprine (%)	3	4	5
Mycophenolate/mycophenolate mofetil (%)	92	90	85
Everolimus (%)	4	4	4
Sirolimus (%)	0	0	0
Prednisolone (%)	99	99	99

Abbreviations: ALT, alanine aminotransferase; BMI, body mass index; eGFR, estimated glomerular filtration rate; IQR, interquartile range; PCP, *Pneumocystis jirovecii*. ^a^ Two atovaquone users (=1%) were also using trimethoprim chronically, whereas the other seven only used atovaquone as prophylactic agent.

**Table 2:** Overview of selected features.

Features with Even *m*/*z* Values				Features with Odd *m*/*z* Values			
*m*/*z*	RT (min)	Rel. Median (%) ^a^	*p* Value	*m*/*z*	RT (min)	Rel. Median (%) ^a^	*p* Value
254.058 ^b^	6.6	49.1	1.0 × 10^−149^	291.144 ^c^	5.5	100.0	6.5 × 10^−189^
258.089	3.6	5.3	3.6 × 10^−142^	307.138	6.0	7.0	3.1 × 10^−126^
270.052	4.4	4.1	7.8 × 10^−106^	307.139	6.9	4.7	6.2 × 10^−107^
270.053	6.5	2.6	1.7 × 10^−70^	307.139	4.8	2.8	2.6 × 10^−122^
296.068	7.8	185.4	3.5 × 10^−306^	357.083	4.3	4.5	5.8 × 10^−116^
300.100	5.1	11.8	1.7 × 10^−158^	357.084	3.2	1.1	3.1 × 10^−90^
312.063	7.1	5.4	5.0 × 10^−143^	453.157	4.1	14.0	5.7 × 10^−115^
312.063	8.5	1.3	3.4 × 10^−120^	453.158	3.2	6.1	2.4 × 10^−132^
312.064	6.0	21.3	2.3 × 10^−133^	467.175	4.8	10.1	2.1 × 10^−131^
430.088	5.4	35.2	9.2 × 10^−173^	483.168	5.2	2.5	5.0 × 10^−83^
430.088	3.3	1.0	2.5 × 10^−142^				
472.099	4.5	4.5	4.4 × 10^−120^				
472.099	6.9	6.2	7.8 × 10^−126^				

Abbreviations: *m*/*z*, mass-to-charge ratio; RT, retention time; rel., relative. ^a^ The median intensity value observed for the feature corresponding to trimethoprim was set at 100%, and all the other median values were expressed relative to this highest value. ^b^ Feature corresponding to sulfamethoxazole, as confirmed using a chemical reference standard (Sigma-Aldrich, Zwijndrecht, The Netherlands, Cat. No. S7507). ^c^ Feature corresponding to trimethoprim, as confirmed using a chemical reference standard (Duchefa Biochemie, Haarlem, The Netherlands, T0154).

**Table 3:** Overview of (putatively) identified sulfamethoxazole metabolites.

Identity ^a,b^	Abbreviation	Molecular Formula	*m*/*z*	RT (min)	Median (IQR; Range) Metabolite Abundance ^c^ in 152 KTRs (%)
Sulfamethoxazole	S	C_10_H_11_N_3_O_3_S	254.06	6.6	16.5 (11.1–20.9; 4.6–34.6)
Isoxazole ring-opened sulfamethoxazole	iro-S	C_10_H_15_N_3_O_3_S	258.09	3.6	1.6 (1.2–2.1; 0.0–6.3)
Hydroxysulfamethoxazole or	S-O or S-HA	C_10_H_11_N_3_O_4_S	270.05	4.4	1.2 (0.7–1.8; 0.2–3.5)
sulfamethoxazole hydroxylamine				6.5	0.8 (0.4–1.4; 0.0–3.5)
Acetylsulfamethoxazole	S-Ac	C_12_H_13_N_3_O_4_S	296.07	7.8	59.2 (56.0–63.1; 39.4–80.6)
Isoxazole ring-opened acetylsulfamethoxazole	iro-S-Ac	C_12_H_17_N_3_O_4_S	300.10	5.1	3.0 (2.2–3.8; 0.1–10.1)
Oxygenated acetylsulfamethoxazole	S-Ac-O	C_12_H_13_N_3_O_5_S	312.06	6.0	5.3 (3.4–7.0; 1.0–24.1)
				7.1	1.4 (0.9–1.9; 0.3–3.5)
				8.5	0.3 (0.2–0.4; 0.0–0.9)
Sulfamethoxazole glucuronide	S-GLU	C_16_H_19_N_3_O_9_S	430.09	3.3	0.2 (0.1–0.2; 0.0–0.5)
				5.4	6.7 (5.2–8.3; 0.8–14.7)
Acetylsulfamethoxazole glucuronide	S-Ac-GLU	C_18_H_21_N_3_O_10_S	472.10	4.5	0.7 (0.5–1.1; 0.2–3.8)
				6.9	1.1 (0.8–1.5; 0.1–5.3)

Abbreviations: IQR, interquartile range; *m*/*z*, mass-to-charge ratio; KTRs, kidney transplant recipients; RT, retention time. ^a^ The only substance whose identity was verified using a chemical reference standard was sulfamethoxazole. All metabolites were putatively identified based upon their spectral similarity to known substances. ^b^ Exemplary extracted ion chromatograms and fragment spectra are shown in Figures S2–S9. ^c^ The median metabolite abundance values presented in the table reflect the median values of the relative quantitative readouts that were calculated by dividing the signal intensity of each individual substance by the sum of the signal intensities of all the substances found per sulfamethoxazole user.

**Table 4:** Overview of (putatively) identified trimethoprim metabolites.

Identity ^a,b^	Abbreviation	Molecular Formula	*m*/*z*	RT (min)	Median (IQR; Range) Metabolite Abundance ^c^ in 156 KTRs (%)
Trimethoprim	T	C_14_H_18_N_4_O_3_	291.14	5.5	74.6 (68.2–79.4; 54.3–90.8)
Oxygenated trimethoprim	T-O	C_14_H_18_N_4_O_4_	307.14	4.8	1.7 (1.3–2.2; 0.6–4.6)
				6.0	4.2 (3.2–5.3; 0.9–7.7)
				6.9	2.9 (2.2–3.8; 0.9–6.5)
Demethyl trimethoprim sulfate	T-DM-SUL	C_13_H_16_N_4_O_6_S	357.08	3.2	0.5 (0.4–0.8; 0.1–15.4)
				4.3	2.4 (1.8–3.4; 0.7–5.5)
Demethyl trimethoprim glucuronide	T-DM-GLU	C_19_H_24_N_4_O_9_	453.16	3.2	2.3 (1.7–3.2; 0.6–5.1)
				4.1	5.7 (4.2–7.3; 1.5–15.6)
Trimethoprim glucuronide	T-GLU	C_20_H_26_N_4_O_9_	467.17	4.8	3.7 (2.8–4.5; 0.5–8.7)
				5.2	0.4 (0.3–0.6; 0.1–1.2)
				5.7	0.1 (0.1–0.1; 0.0–1.3)
Oxygenated trimethoprim glucuronide	T-O-GLU	C_20_H_26_N_4_O_10_	483.17	5.2	0.9 (0.6–1.3; 0.1–3.6)

Abbreviations: IQR, interquartile range; *m*/*z*, mass-to-charge ratio; KTRs, kidney transplant recipients; RT, retention time. ^a^ The only substance whose identity was verified using a chemical reference standard was trimethoprim. All metabolites were putatively identified based upon their spectral similarity to known substances. ^b^ Exemplary extracted ion chromatograms and fragment spectra are shown in Figures S10–S15. ^c^ The median metabolite abundance values presented in the table reflect the median values of the relative quantitative readouts that were calculated by dividing the signal intensity of each individual substance by the sum of the signal intensities of all the substances found per trimethoprim user.

## Data Availability

All the pharmacometabolomics data have been deposited in an open-access data repository, which can be found at https://doi.org/10.26037/yareta:64ruex2sxff5nenyfyexurzs3m (as sub-study 5).

## Supplementary Materials

The following supporting information can be downloaded at https://www.mdpi.com/article/10.3390/metabo15070473/s1: Data S1. Computational details of quantum chemistry simulations. Figure S1. Exemplary spectral library matching-based identification of sulfamethoxazole and trimethoprim, which were identified in the urine of a kidney transplant recipient who declared their usage of this drug combination. Figure S2. MS1-level extracted ion chromatogram, SWATH/MS fragment spectrum, and product ion scan fragment spectrum of sulfamethoxazole observed in the urine of a human cotrimoxazole user as well as the product ion scan fragment spectrum of a sulfamethoxazole reference standard. Figure S3. MS1-level extracted ion chromatogram, SWATH/MS fragment spectrum, and product ion scan fragment spectrum of an acetylated version of sulfamethoxazole observed in the urine of a human cotrimoxazole user. Figure S4. MS1-level extracted ion chromatogram, SWATH/MS fragment spectra, and product ion scan fragment spectra of two oxidized versions of sulfamethoxazole observed in the urine of a human cotrimoxazole user. Figure S5. MS1-level extracted ion chromatogram, SWATH/MS fragment spectra, and product ion scan fragment spectra of three oxidized and acetylated versions of sulfamethoxazole observed in the urine of a human cotrimoxazole user. Figure S6. MS1-level extracted ion chromatogram, SWATH/MS fragment spectra, and product ion scan fragment spectra of two glucuronidated versions of sulfamethoxazole observed in the urine of a human cotrimoxazole user. Figure S7. MS1-level extracted ion chromatogram, SWATH/MS fragment spectra, and product ion scan fragment spectra of two acetylated and glucuronidated versions of sulfamethoxazole observed in the urine of a human cotrimoxazole user. Figure S8. MS1-level extracted ion chromatogram, SWATH/MS fragment spectrum, and product ion scan fragment spectrum of an unknown sulfamethoxazole metabolite or impurity (metabolite) which could possibly correspond to an isoxazole ring-opened version of sulfamethoxazole observed in the urine of a human cotrimoxazole user. Figure S9. MS1-level extracted ion chromatogram, SWATH/MS fragment spectrum, and product ion scan fragment spectrum of an unknown sulfamethoxazole metabolite or impurity (metabolite) which could possibly correspond to an acetylated and isoxazole ring-opened version of sulfamethoxazole observed in the urine of a human cotrimoxazole user. Figure S10. MS1-level extracted ion chromatogram, SWATH/MS fragment spectrum, and product ion scan fragment spectrum of trimethoprim observed in the urine of a human cotrimoxazole user as well as the product ion scan fragment spectrum of a trimethoprim reference standard. Figure S11. MS1-level extracted ion chromatogram, SWATH/MS fragment spectra, and product ion scan fragment spectra of three oxidized versions of trimethoprim observed in the urine of a human cotrimoxazole user. Figure S12. MS1-level extracted ion chromatogram, SWATH/MS fragment spectra, and product ion scan fragment spectra of two demethylated and glucuronidated versions of trimethoprim observed in the urine of a human cotrimoxazole user. Figure S13. MS1-level extracted ion chromatogram, SWATH/MS fragment spectra, and product ion scan fragment spectra of two demethylated and sulfated versions of trimethoprim observed in the urine of a human cotrimoxazole user. Figure S14. MS1-level extracted ion chromatogram, SWATH/MS fragment spectra, and product ion scan fragment spectra of three glucuronidated versions of trimethoprim observed in the urine of a human cotrimoxazole user. Figure S15. MS1-level extracted ion chromatogram, SWATH/MS fragment spectrum, and product ion scan fragment spectrum of an oxidized and glucuronidated version of trimethoprim observed in the urine of a human cotrimoxazole user. Figure S16. Putative human metabolism of sulfamethoxazole based on this study’s findings. Figure S17. Putative human metabolism of trimethoprim based on this study’s findings. Table S1. Overview of MarkerView data (pre)processing settings. Table S2. Overview of representative (candidate) fragments of sulfamethoxazole, as depicted in Figure S2. Table S3. Overview of representative (candidate) fragments of trimethoprim, as depicted in Figure S10.

## Abbreviations

The following abbreviations are used in this manuscript:

ALT	alanine aminotransferase
BMI	body mass index
CKD-EPI	chronic kidney disease epidemiology collaboration
CVID	common variable immune deficiency
DIA	data-independent acquisition
eGFR	estimated glomerular filtration rate
HRMS	high-resolution mass spectrometry
IQR	interquartile range
KTRs	kidney transplant recipients
LC	liquid chromatography
*m*/*z*	mass-to-charge ratio
NCT	national clinical trial
PCP	Pneumocystis jirovecii
PMx	pharmacometabolomics
PNEC	predicted no-effect concentration
RT	retention time
SWATH	sequential window acquisition of all theoretical fragment ion spectra
TOF	time-of-flight
