# Supplementary material for: Pharmacometabolomics Study of Sulfamethoxazole and Trimethoprim in Kidney Transplant Recipients: Real-World Metabolism and Urinary Excretion
Source: Metabolites. 2025 Jul 11;15(7):473. doi: 10.3390/metabo15070473 (PMC12299322; doi:10.3390/metabo15070473)
Supplement: Supplementary file 1 [file metabolites-15-00473-s001.zip › metabolites-3729066-supplementary.pdf]

Pharmacometabolomics study of sulfamethoxazole and trimethoprim in kidney transplant recipients: *real-world metabolism and urinary excretion*

Marieke A.J. Hof [1,†], Hessel de Haan [2,†], Stepan Stepanovic [3,4,†], Stephan J.L. Bakker [5], Eelko Hak [2], Gérard Hopfgartner [3], Frank Klont [2,6,*], On Behalf Of The TransplantLines Investigators [7]

[1] Department of Analytical Biochemistry, Groningen Research Institute of Pharmacy, University of Groningen, Groningen, the Netherlands
[2] Unit of PharmacoTherapy, -Epidemiology & -Economics, Groningen Research Institute of Pharmacy, University of Groningen, Groningen, the Netherlands
[3] Life Sciences Mass Spectrometry, Department of Inorganic and Analytical Chemistry, University of Geneva, Genève, Switzerland
[4] Institute of Chemistry, Technology and Metallurgy, University of Belgrade, Belgrade, Serbia
[5] Division of Nephrology, Department of Internal Medicine, University Medical Center Groningen, University of Groningen, Groningen, the Netherlands
[6] Department of Clinical Pharmacy and Pharmacology, University Medical Center Groningen, University of Groningen, Groningen, the Netherlands
[7] Group of Authors on Behalf of the Transplant Lines Biobank and Cohort Study, University Medical Center Groningen, University of Groningen, Hanzeplein 1, 9700 RB Groningen, The Netherlands
[*] Author to whom correspondence should be addressed (frank.klont@rug.nl).
[†] These authors contributed equally to this work.

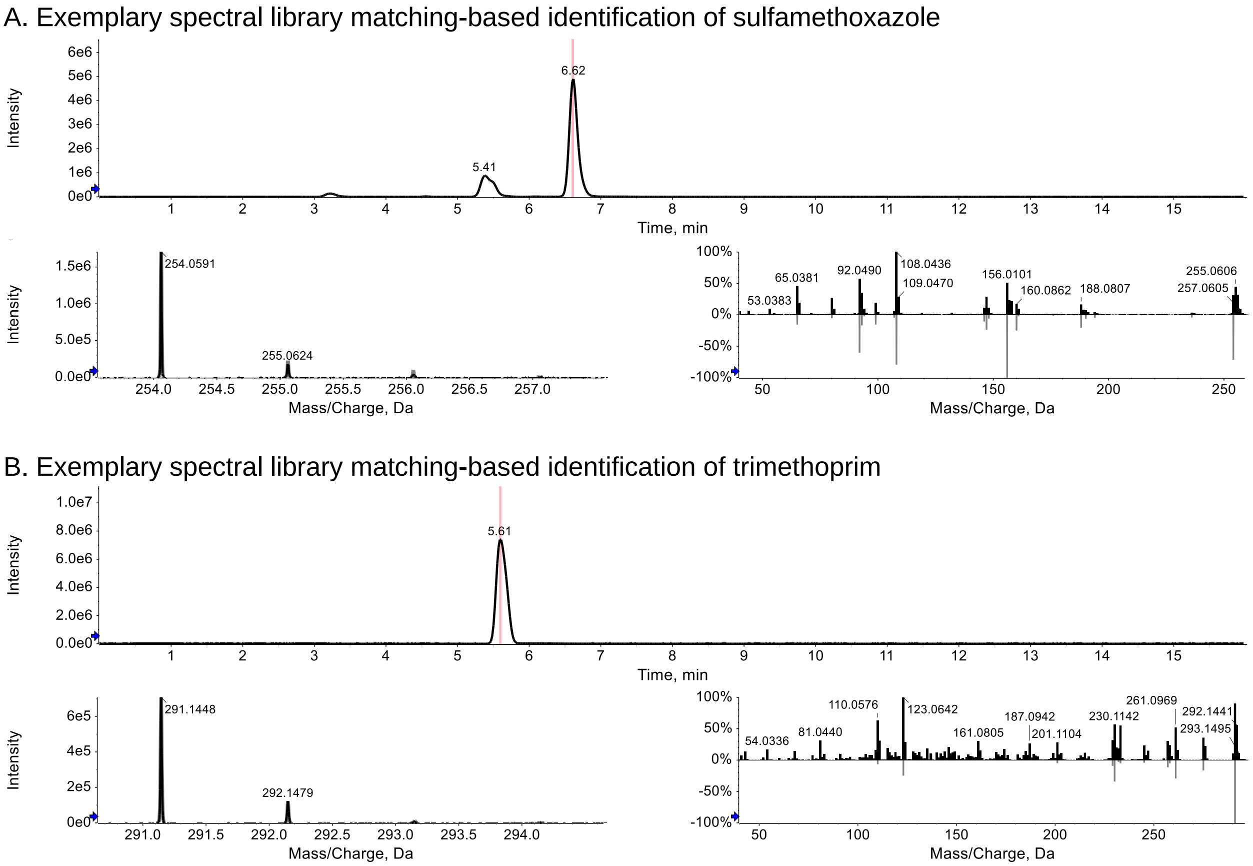

Figure S1. Exemplary spectral library matching-based identification of (**A**) sulfamethoxazole (PubChem Compound IDentification (CID) number 5329) and (**B**) trimethoprim (CID 5578) which were observed in the urine of a kidney transplant recipient who declared usage of this drug combination. Regarding the data obtained and presented, these concern level 2 metabolite identifications according to the Metabolomics Standards Initiative (Sumner LW, Amberg A, Barrett D, *et al*. Proposed minimum reporting standards for chemical analysis Chemical Analysis Working Group (CAWG) Metabolomics Standards Initiative (MSI). Metabolomics 2007; 3: 211–221) for which we employed a commercial spectral library (SCIEX 'Forensic', version 1.1) and a commercial software tool (SCIEX PeakView, version 2.2.0.11391) using previously-published settings (Klont F, Kremer D, Gomes Neto AW, *et al*. Metabolomics data complemented drug use information in epidemiological databases: pilot study of potential kidney donors. Journal of Clinical Epidemiology 2021; 135: 10-16).

Table S1. Overview of MarkerView data (pre)processing settings.

Setting	Value
Feature finding:	
Experiment	MS1
Minimum retention time	0.50 min
Maximum retention time	16.00 min
Subtraction offset	15 scans
Subtraction multiplication factor	1.3
Noise threshold	5
Minimum spectral peak width	5 ppm
Minimum retention time peak width	5 scans
Assign charge states	Enabled
Feature alignment:	
Retention time tolerance	0.50 min
Mass tolerance	0.01 Da
Feature filtering:	
Maximum number of peaks	8,000,000*
Remove peaks in < N samples	Disabled
Isotope filtering	Disabled
Intensity threshold	5
Use exclusion list	Disabled
Retention time filtering	Disabled
Use area integrated from raw data, not from original peak finding	Disabled
Principle component analysis:	
PCA preprocessing - Weighting	None
PCA preprocessing - Scaling	Pareto
Perform PCA-DA (supervised)	Disabled
T-test:	
Samples per group for “first to last” comparison	Disabled
Use Welch t-test	Disabled

*: This parameter was set high enough to prevent peaks from getting filtered at this stage.

A. MS1-level extracted ion chromatogram (m/z 254.0594 ± 0.0025) of an exemplary cotrimoxazole user

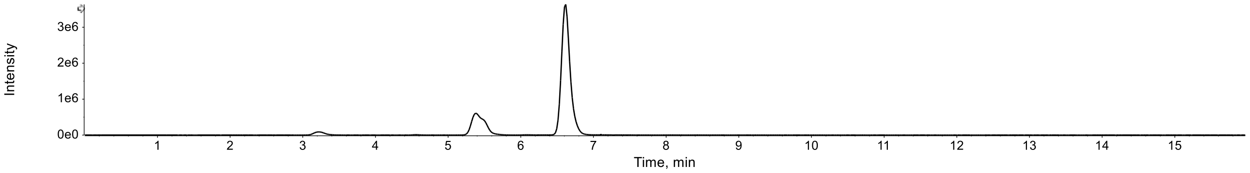

B. SWATH/MS fragment spectrum of the peak at 6.6 min.

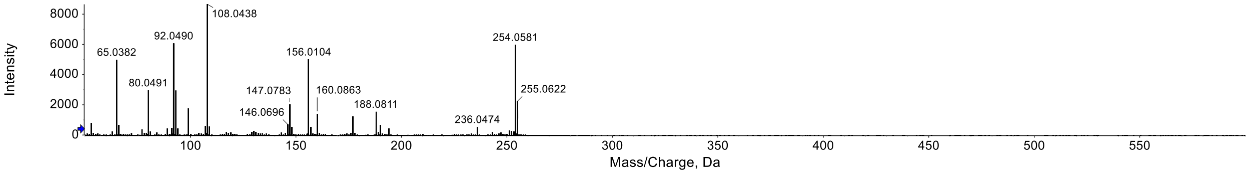

C. Product ion scan fragment spectrum of the peak at 6.6 min.

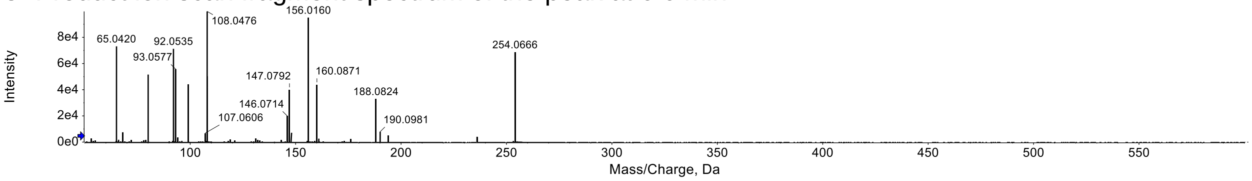

D. Product ion scan fragment spectrum of a sulfamethoxazole chemical reference standard

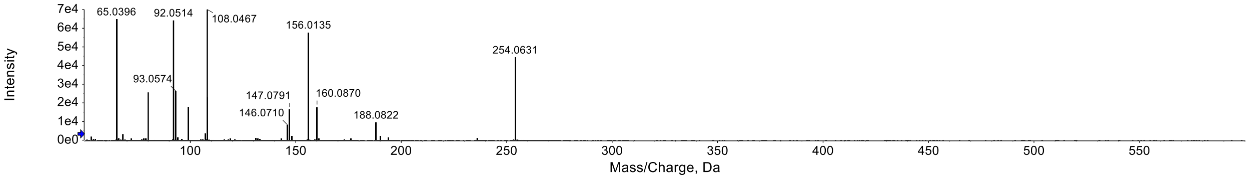

Figure S2. (**A**) MS1-level extracted ion chromatogram, (**B**) SWATH/MS fragment spectrum, and (**C**) product ion scan fragment spectrum of sulfamethoxazole observed in urine of a human cotrimoxazole user as well as (**D**) product ion scan fragment spectrum of a sulfamethoxazole reference standard (Sigma-Aldrich, Cat. No. S7507). The substance featured in this figure (surely being sulfamethoxazole, CID 5329) reflects a 'level 1' identification in terms of the classification proposed by the Metabolomics Standards Initiative (Sumner LW, Amberg A, Barrett D, *et al*. Proposed minimum reporting standards for chemical analysis Chemical Analysis Working Group (CAWG) Metabolomics Standards Initiative (MSI). Metabolomics 2007; 3: 211–221).

Table S2. Overview of representative (candidate) fragments of sulfamethoxazole, as depicted in Figure S2, pane B. Regarding the candidate fragments, these were derived utilizing the ‘Fragment Pane’ module in SCIEX PeakView (version 2.2.0.11391), drawing from previously annotated and published fragment spectra.

Signal	Candidate fragment	Candidate molecular formula	Expected m/z	S<br>Observed m/z (mass error)
Fragment 92	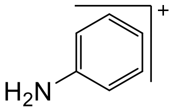	C_6H_6N	92.0500	92.0490 (-11 ppm)
Fragment 108	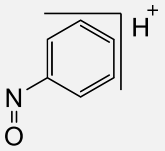	C_6H_6NO	108.0449	108.0438 (-11 ppm)
Fragment 156	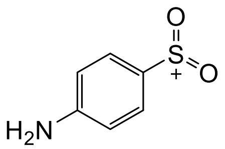	$C_6H_6NO_2S$	156.0119	156.0104 (-10 ppm)
Residual Precursor	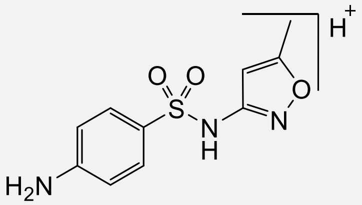	$C_{10}H_{12}N_3O_3S$	254.0599	254.0581 (-7 ppm)

A. MS1-level extracted ion chromatogram (m/z 296.0700 ± 0.0025) of an exemplary cotrimoxazole user

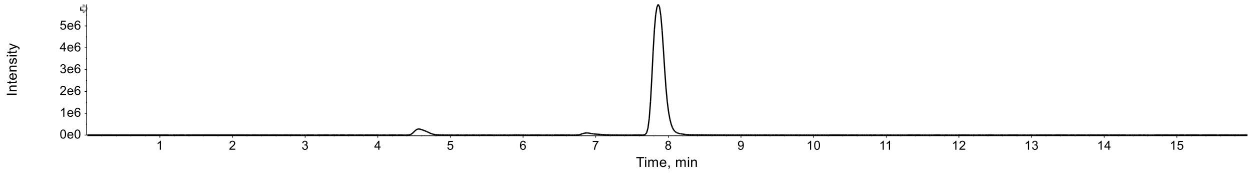

B. SWATH/MS fragment spectrum of the peak at 7.8 min.

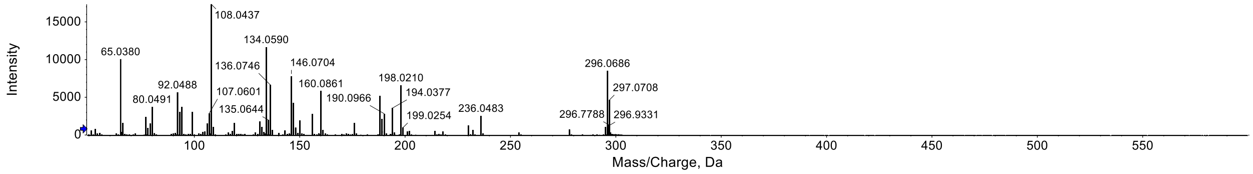

C. Product ion scan fragment spectrum of the peak at 7.8 min.

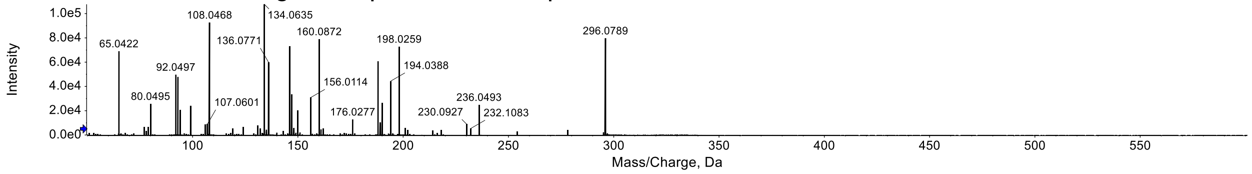

Figure S3. (**A**) MS1-level extracted ion chromatogram, (**B**) SWATH/MS fragment spectrum, and (**C**) product ion scan fragment spectrum of an acetylated version of sulfamethoxazole (+42 Da) observed in urine of a human cotrimoxazole user. The substance featured in this figure (possibly being N-acetylsulfamethoxazole, CID 65280) reflects a 'level 3' identification in terms of the classification proposed by the Metabolomics Standards Initiative (Sumner LW, Amberg A, Barrett D, *et al*. Proposed minimum reporting standards for chemical analysis Chemical Analysis Working Group (CAWG) Metabolomics Standards Initiative (MSI). Metabolomics 2007; 3: 211–221).

A. MS1-level extracted ion chromatogram (m/z 270.0543 ± 0.0025) of an exemplary cotrimoxazole user

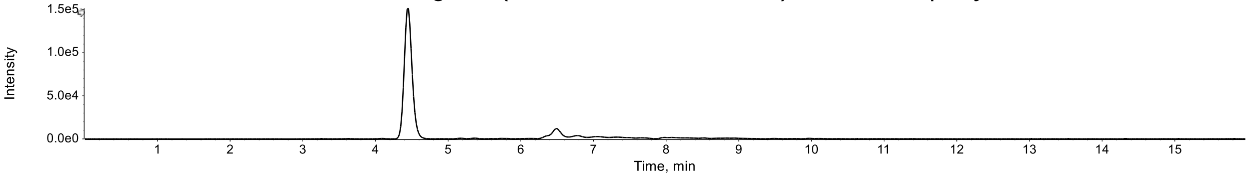

B. SWATH/MS fragment spectrum of the peak at 4.5 min.

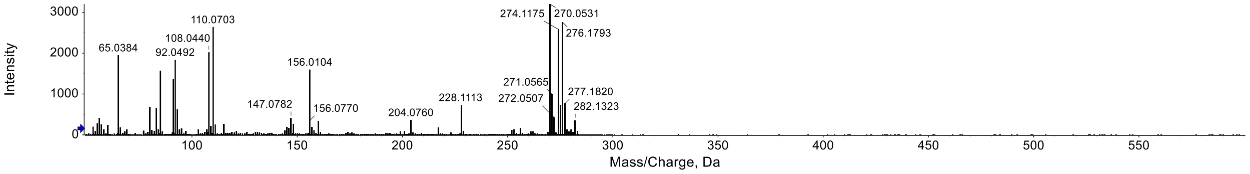

C. SWATH/MS fragment spectrum of the peak at 6.5 min.

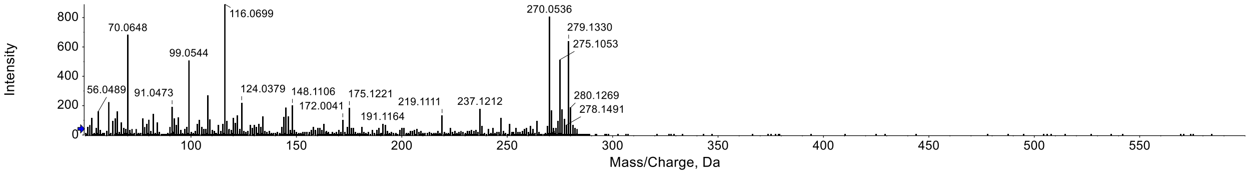

D. Product ion scan fragment spectrum of the peak at 4.5 min.

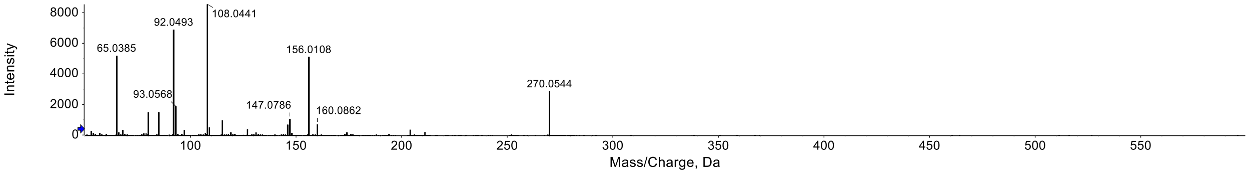

E. Product ion scan fragment spectrum of the peak at 6.5 min.

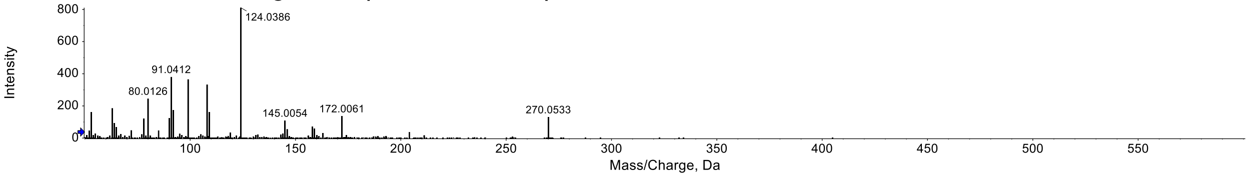

Figure S4. (**A**) MS1-level extracted ion chromatogram, (**B**-**C**) SWATH/MS fragment spectra, and (**D**-**E**) product ion scan fragment spectra of two oxidized versions of sulfamethoxazole (+16 Da) observed in urine of a human cotrimoxazole user. The substances featured in this figure (possibly being the two known metabolites 5-hydroxysulfamethoxazole, CID 10355717, and sulfamethoxazole hydroxylamine, CID 114821) reflect ‘level 3’ identifications in terms of the classification proposed by the Metabolomics Standards Initiative (Sumner LW, Amberg A, Barrett D, *et al*. Proposed minimum reporting standards for chemical analysis Chemical Analysis Working Group (CAWG) Metabolomics Standards Initiative (MSI). Metabolomics 2007; 3: 211–221).

A. MS1-level extracted ion chromatogram (m/z 312.0649 ± 0.0025) of an exemplary cotrimoxazole user

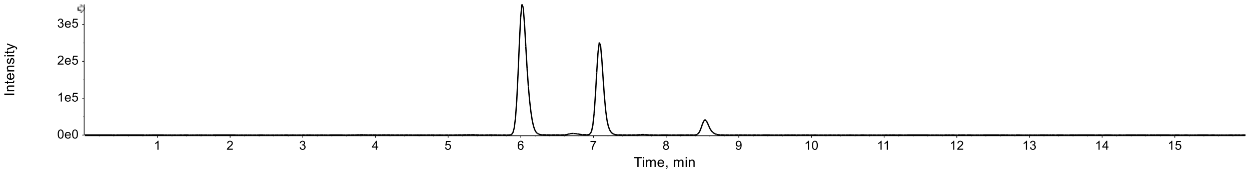

B. SWATH/MS fragment spectrum of the peak at 6.0 min.

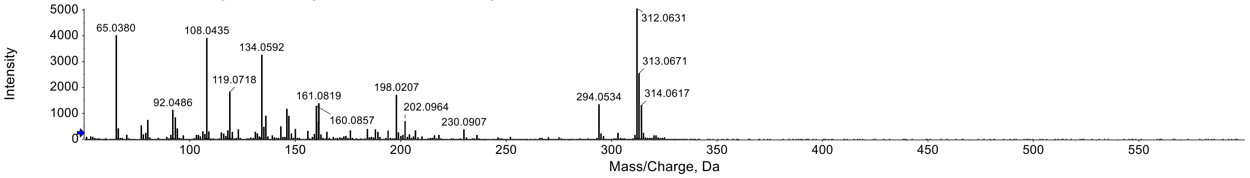

C. SWATH/MS fragment spectrum of the peak at 7.1 min.

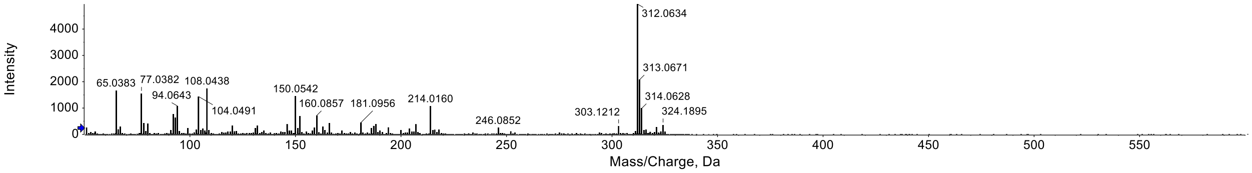

D. SWATH/MS fragment spectrum of the peak at 8.5 min.

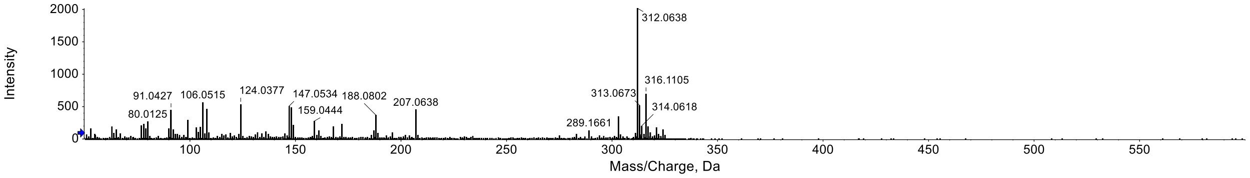

E. Product ion scan fragment spectrum of the peak at 6.0 min.

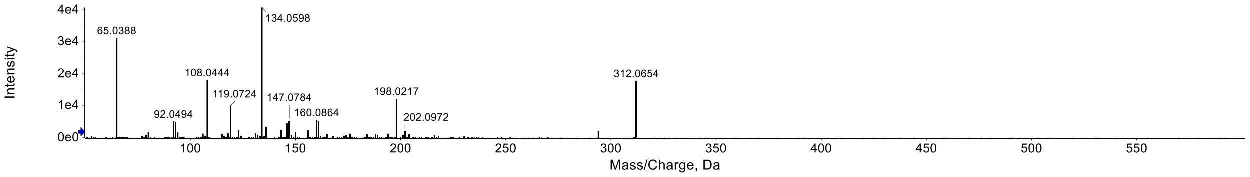

F. Product ion scan fragment spectrum of the peak at 7.1 min.

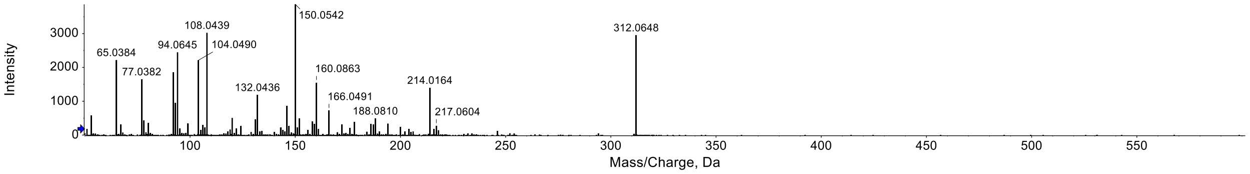

G. Product ion scan fragment spectrum of the peak at 8.5 min.

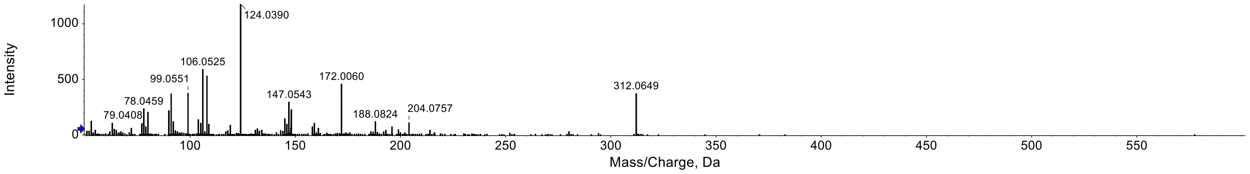

Figure S5. (**A**) MS1-level extracted ion chromatogram, (**B**-**D**) SWATH/MS fragment spectra, and (**E**-**G**) product ion scan fragment spectra of three oxidized and acetylated versions of sulfamethoxazole (+16, +42 Da) observed in urine of a human cotrimoxazole user. The substances featured in this figure (possibly being N4-acetyl-5-OH-sulfamethoxazole, CID 10335611, and two other acetylated and oxidized versions of sulfamethoxazole, for which no putative InChI identifiers are provided due to the uncertain positions of the added oxygen atoms) reflect 'level 3' identifications in terms of the classification proposed by the Metabolomics Standards Initiative (Sumner LW, Amberg A, Barrett D, *et al*. Proposed minimum reporting standards for chemical analysis Chemical Analysis Working Group (CAWG) Metabolomics Standards Initiative (MSI). Metabolomics 2007; 3: 211–221).

A. MS1-level extracted ion chromatogram (m/z 430.0915 ± 0.0025) of an exemplary cotrimoxazole user

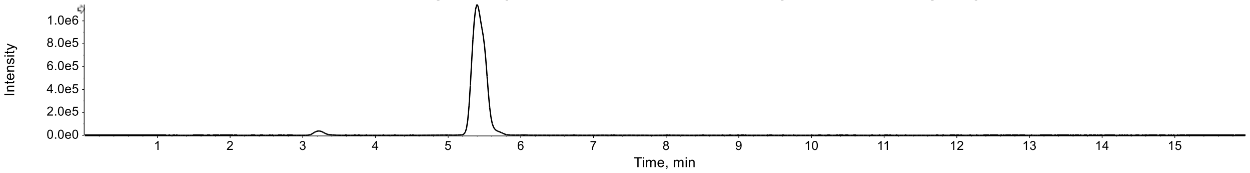

B. SWATH/MS fragment spectrum of the peak at 3.2 min.

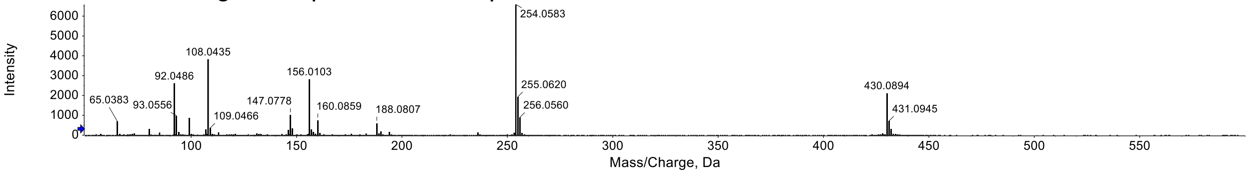

C. SWATH/MS fragment spectrum of the peak at 5.4 min.

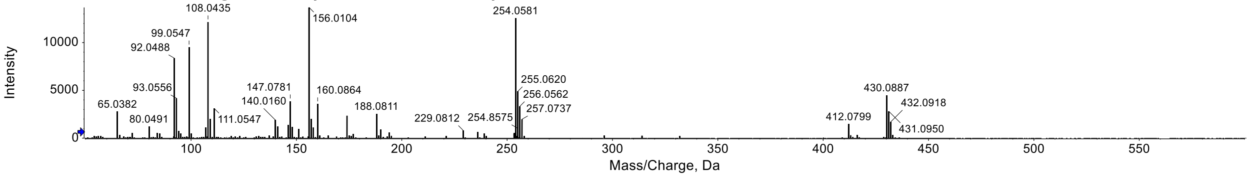

D. Product ion scan fragment spectrum of the peak at 3.2 min.

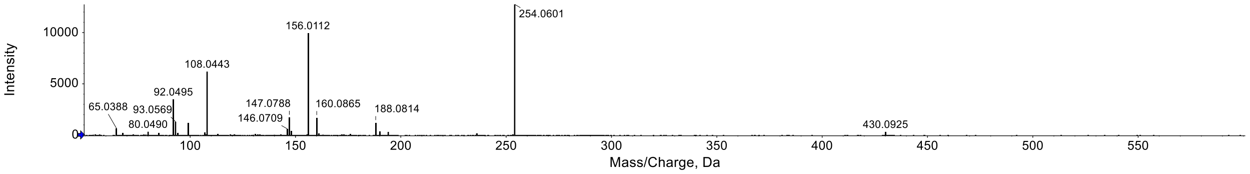

E. Product ion scan fragment spectrum of the peak at 5.4 min.

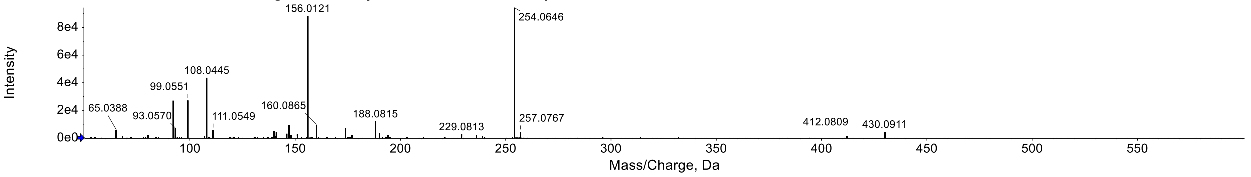

Figure S6. (**A**) MS1-level extracted ion chromatogram, (**B**-**C**) SWATH/MS fragment spectra, and (**D**-**E**) product ion scan fragment spectra of two glucuronidated versions of sulfamethoxazole (+176 Da) observed in urine of a human cotrimoxazole user. The substances featured in this figure (possibly being sulfamethoxazole N1-Glucuronide, CID 75213081, and another glucuronidated version of sulfamethoxazole, for which no putative InChI identifier is provided due to the uncertain position of the glucuronide moiety) reflect 'level 3' identifications in terms of the classification proposed by the Metabolomics Standards Initiative (Sumner LW, Amberg A, Barrett D, *et al*. Proposed minimum reporting standards for chemical analysis Chemical Analysis Working Group (CAWG) Metabolomics Standards Initiative (MSI). Metabolomics 2007; 3: 211–221).

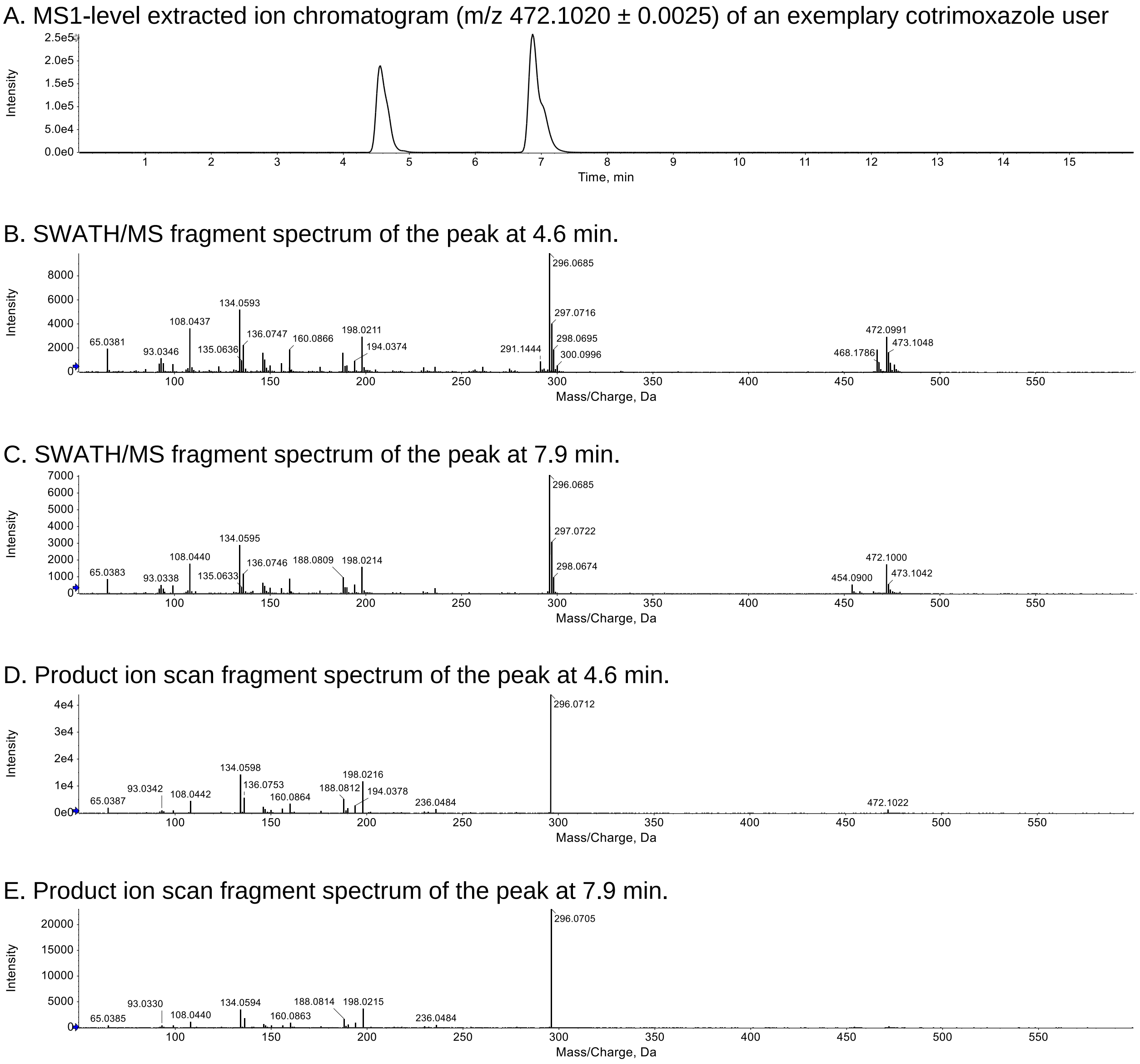

Figure S7. (**A**) MS1-level extracted ion chromatogram, (**B**-**C**) SWATH/MS fragment spectra, and (**D**-**E**) product ion scan fragment spectra of two acetylated and glucuronidated versions of sulfamethoxazole (+42, +176 Da) observed in urine of a human cotrimoxazole user. The substances featured in this figure (possibly being two different acetylated and glucuronidated versions of sulfamethoxazole, for which no putative InChI identifiers are provided due to the uncertain positions of the acetyl and glucuronide moieties) reflect 'level 3' identifications in terms of the classification proposed by the Metabolomics Standards Initiative (Sumner LW, Amberg A, Barrett D, *et al*. Proposed minimum reporting standards for chemical analysis Chemical Analysis Working Group (CAWG) Metabolomics Standards Initiative (MSI). Metabolomics 2007; 3: 211–221).

A. MS1-level extracted ion chromatogram (m/z 258.0907 ± 0.0025) of an exemplary cotrimoxazole user

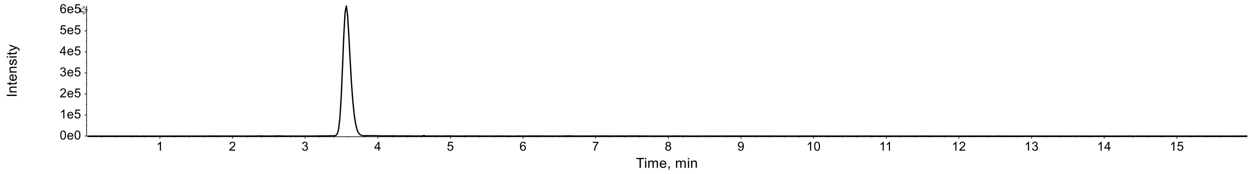

B. SWATH/MS fragment spectrum of the peak at 3.6 min.

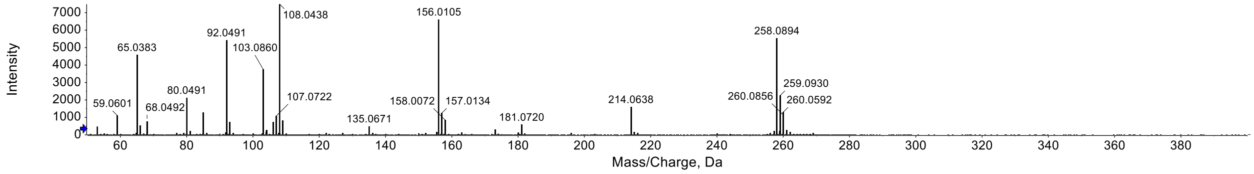

C. Product ion scan fragment spectrum of the peak at 3.6 min.

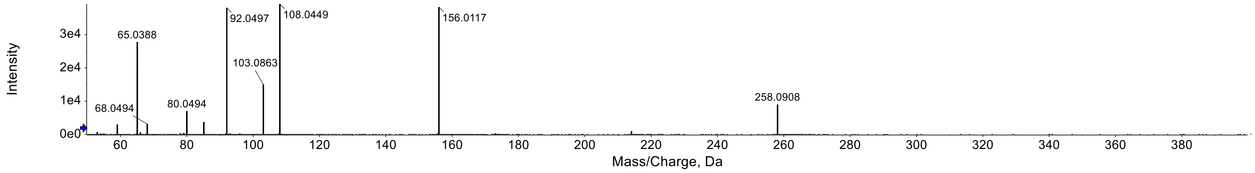

Figure S8. (**A**) MS1-level extracted ion chromatogram, (**B**) SWATH/MS fragment spectrum, and (**C**) product ion scan fragment spectrum of an unknown sulfamethoxazole metabolite or impurity (metabolite) which could possibly correspond to an isoxazole ring-opened version of sulfamethoxazole observed in urine of a human cotrimoxazole user. The substance featured in this figure (possibly being an isoxazole ring-opened version of sulfamethoxazole, see Supplementary Data S1) reflects a ‘level 3’ identification in terms of the classification proposed by the Metabolomics Standards Initiative (Sumner LW, Amberg A, Barrett D, *et al*. Proposed minimum reporting standards for chemical analysis Chemical Analysis Working Group (CAWG) Metabolomics Standards Initiative (MSI). Metabolomics 2007; 3: 211–221).

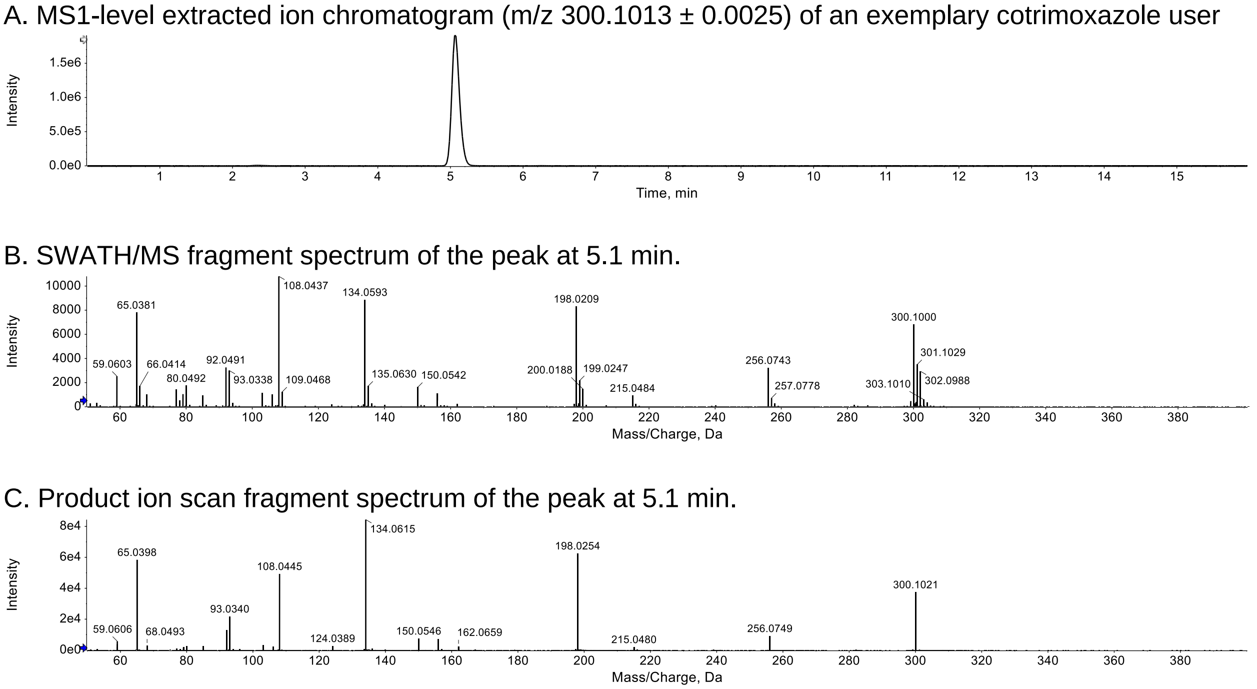

Figure S9. (**A**) MS1-level extracted ion chromatogram, (**B**) SWATH/MS fragment spectrum, and (**C**) product ion scan fragment spectrum of an unknown sulfamethoxazole metabolite or impurity (metabolite) which could possibly correspond to an acetylated and isoxazole ring-opened version of sulfamethoxazole observed in urine of a human cotrimoxazole user. The substance featured in this figure (possibly being an acetylated and isoxazole ring-opened version of sulfamethoxazole, see Supplementary Data S1) reflects a 'level 3' identification in terms of the classification proposed by the Metabolomics Standards Initiative (Sumner LW, Amberg A, Barrett D, *et al*. Proposed minimum reporting standards for chemical analysis Chemical Analysis Working Group (CAWG) Metabolomics Standards Initiative (MSI). Metabolomics 2007; 3: 211–221).

A. MS1-level extracted ion chromatogram (m/z 291.1452 ± 0.0025) of an exemplary cotrimoxazole user

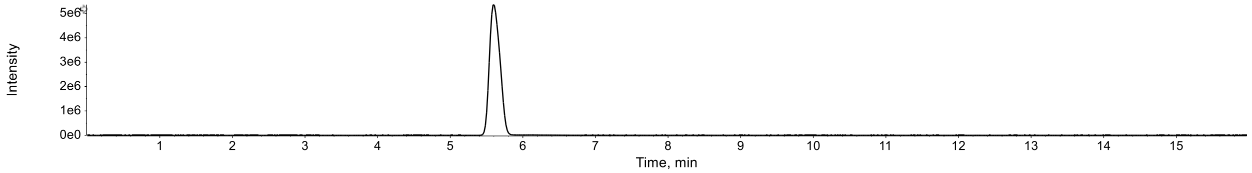

B. SWATH/MS fragment spectrum of the peak at 5.6 min.

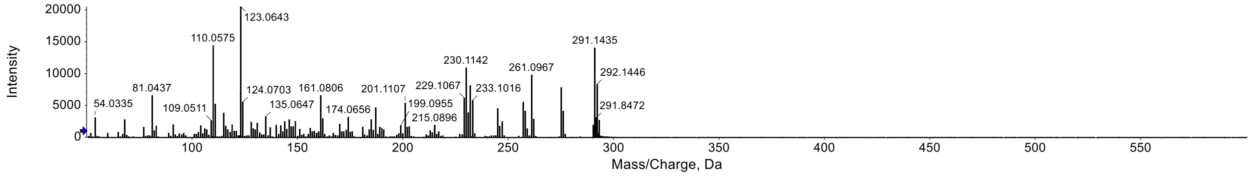

C. Product ion scan fragment spectrum of the peak at 5.6 min.

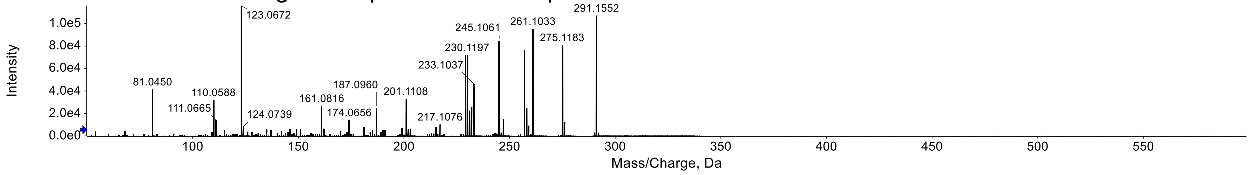

D. Product ion scan fragment spectrum of a trimethoprim chemical reference standard

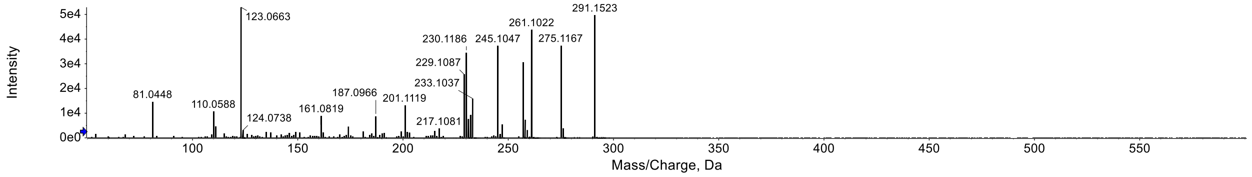

Figure S10. (**A**) MS1-level extracted ion chromatogram, (**B**) SWATH/MS fragment spectrum, and (**C**) product ion scan fragment spectrum of trimethoprim observed in urine of a human cotrimoxazole user as well as (**D**) product ion scan fragment spectrum of a trimethoprim reference standard (Duchefa Biochemie, T0154). The substance featured in this figure (surely being trimethoprim, CID 5578) reflects a 'level 1' identification in terms of the classification proposed by the Metabolomics Standards Initiative (Sumner LW, Amberg A, Barrett D, *et al*. Proposed minimum reporting standards for chemical analysis Chemical Analysis Working Group (CAWG) Metabolomics Standards Initiative (MSI). Metabolomics 2007; 3: 211–221).

Table S3. Overview of representative (candidate) fragments of trimethoprim, as depicted in Figure S10, pane B. Regarding the candidate fragments, these were derived utilizing the ‘Fragment Pane’ module in SCIEX PeakView (version 2.2.0.11391), drawing from previously annotated and published fragment spectra.

				T
**Signal**	**Candidate fragment**	**Candidate molecular formula**	**Expected m/z**	**Observed m/z (mass error)**
Fragment 110	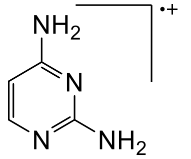	$C_4H_6N_4$	110.0592	110.0575 (-16 ppm)
Fragment 123	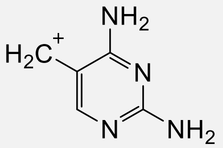	$C_5H_7N_4$	123.0671	123.0643 (-23 ppm)
Fragment 229	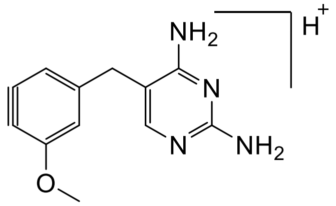	$C_{12}H_{13}N_4O$	229.1089	229.1067 (-10 ppm)
Fragment 230	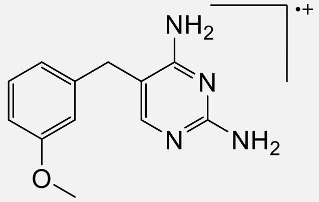	$C_{12}H_{14}N_4O$	230.1168	230.1142 (-11 ppm)
Fragment 261	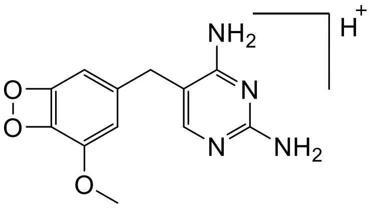	$C_{12}H_{13}N_4O_3$	261.0988	261.0967 (-8 ppm)
Fragment 275	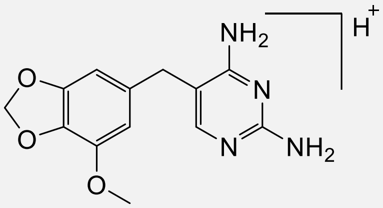	$C_{13}H_{15}N_4O_3$	275.1144	275.1114 (-11 ppm)
Residual Precursor	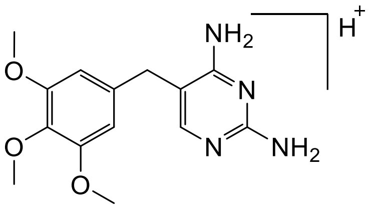	$C_{14}H_{19}N_4O_3$	291.1457	291.1435 (-8 ppm)

A. MS1-level extracted ion chromatogram (m/z 307.1401 ± 0.0025) of an exemplary cotrimoxazole user

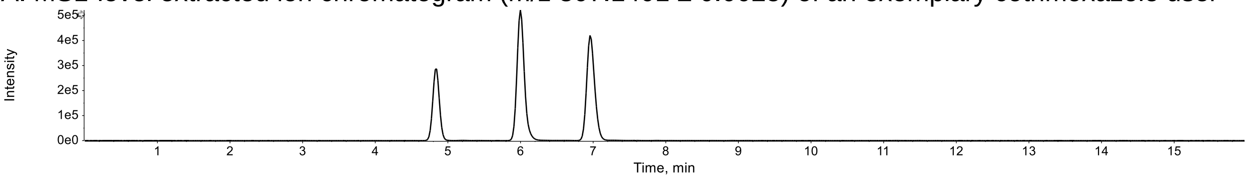

B. SWATH/MS fragment spectrum of the peak at 4.8 min.

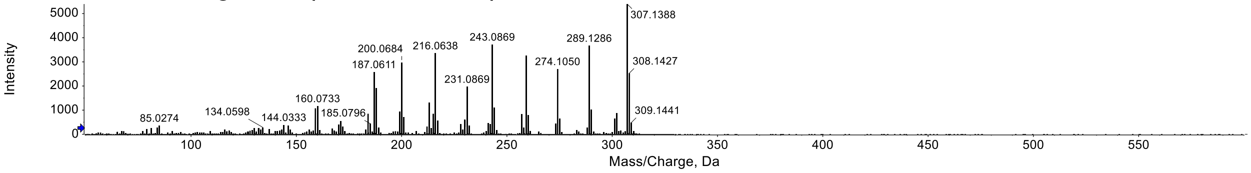

C. SWATH/MS fragment spectrum of the peak at 6.0 min.

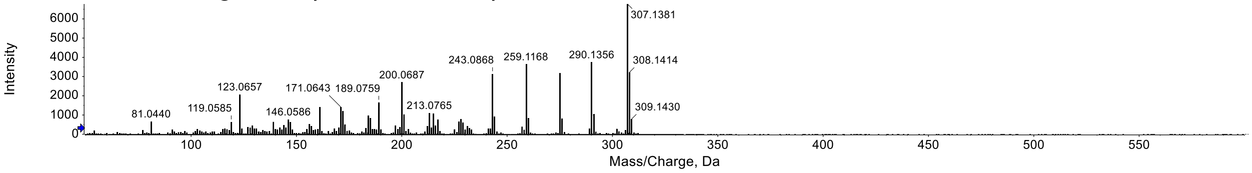

D. SWATH/MS fragment spectrum of the peak at 7.0 min.

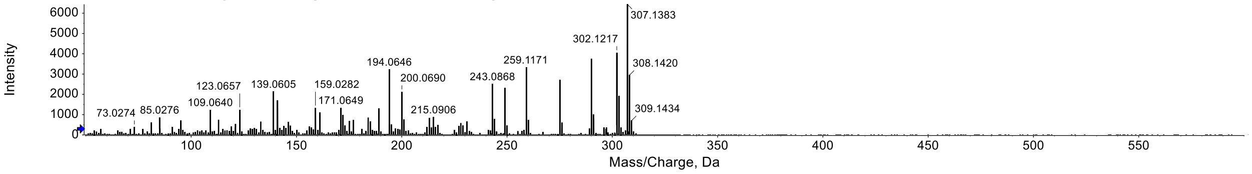

E. Product ion scan fragment spectrum of the peak at 4.8 min.

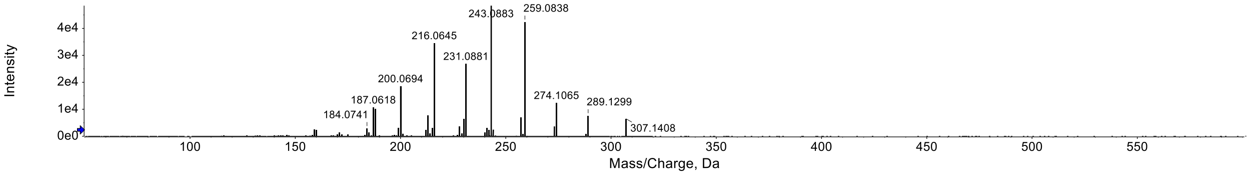

F. Product ion scan fragment spectrum of the peak at 6.0 min.

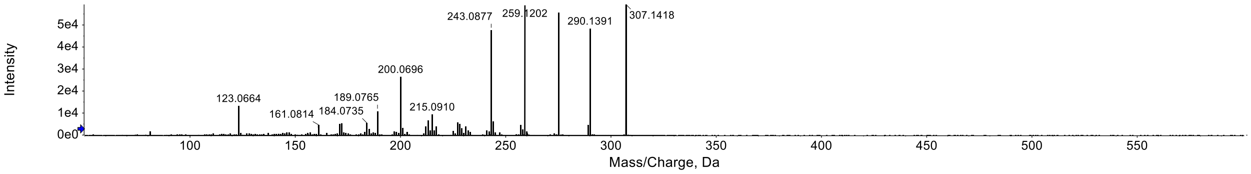

G. Product ion scan fragment spectrum of the peak at 7.0 min.

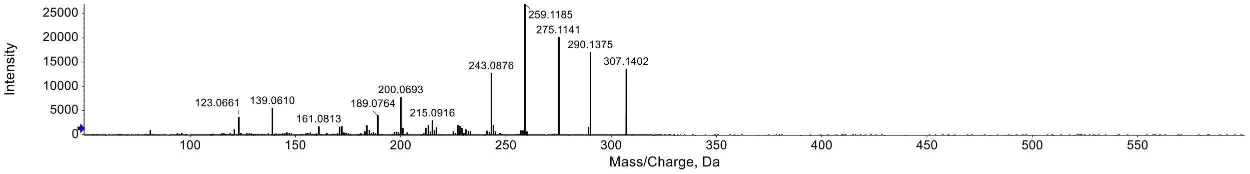

Figure S11. (**A**) MS1-level extracted ion chromatogram, (**B**-**D**) SWATH/MS fragment spectra, and (**E**-**G**) product ion scan fragment spectra of three oxidized versions of trimethoprim (+16 Da) observed in urine of a human cotrimoxazole user. The substances featured in this figure (possibly being alpha-hydroxytrimethoprim, CID 44362947, trimethoprim 1-oxide, CID 5244071, and trimethoprim 3-oxide, CID 23278278) reflect 'level 3' identifications in terms of the classification proposed by the Metabolomics Standards Initiative (Sumner LW, Amberg A, Barrett D, *et al*. Proposed minimum reporting standards for chemical analysis Chemical Analysis Working Group (CAWG) Metabolomics Standards Initiative (MSI). Metabolomics 2007; 3: 211–221).

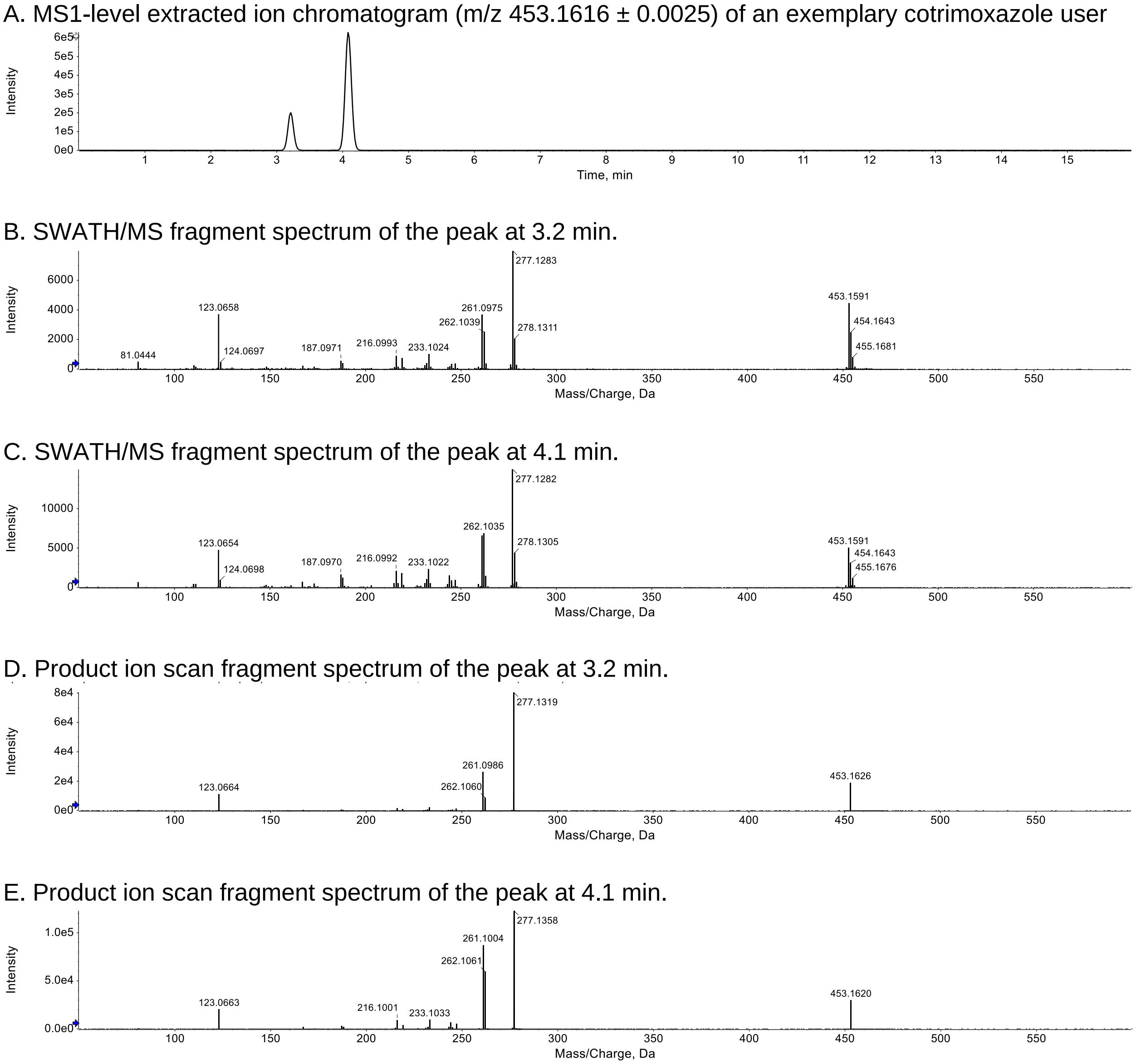

Figure S12. (**A**) MS1-level extracted ion chromatogram, (**B**-**C**) SWATH/MS fragment spectra, and (**D**-**E**) product ion scan fragment spectra of two demethylated and glucuronidated versions of trimethoprim (-14, +176 Da) observed in urine of a human cotrimoxazole user. The substances featured in this figure (possibly being 3-desmethyl trimethoprim glucuronide, CID 169439591, and 4-desmethyl trimethoprim glucuronide, CID 169439592) reflect 'level 3' identifications in terms of the classification proposed by the Metabolomics Standards Initiative (Sumner LW, Amberg A, Barrett D, *et al*. Proposed minimum reporting standards for chemical analysis Chemical Analysis Working Group (CAWG) Metabolomics Standards Initiative (MSI). Metabolomics 2007; 3: 211–221).

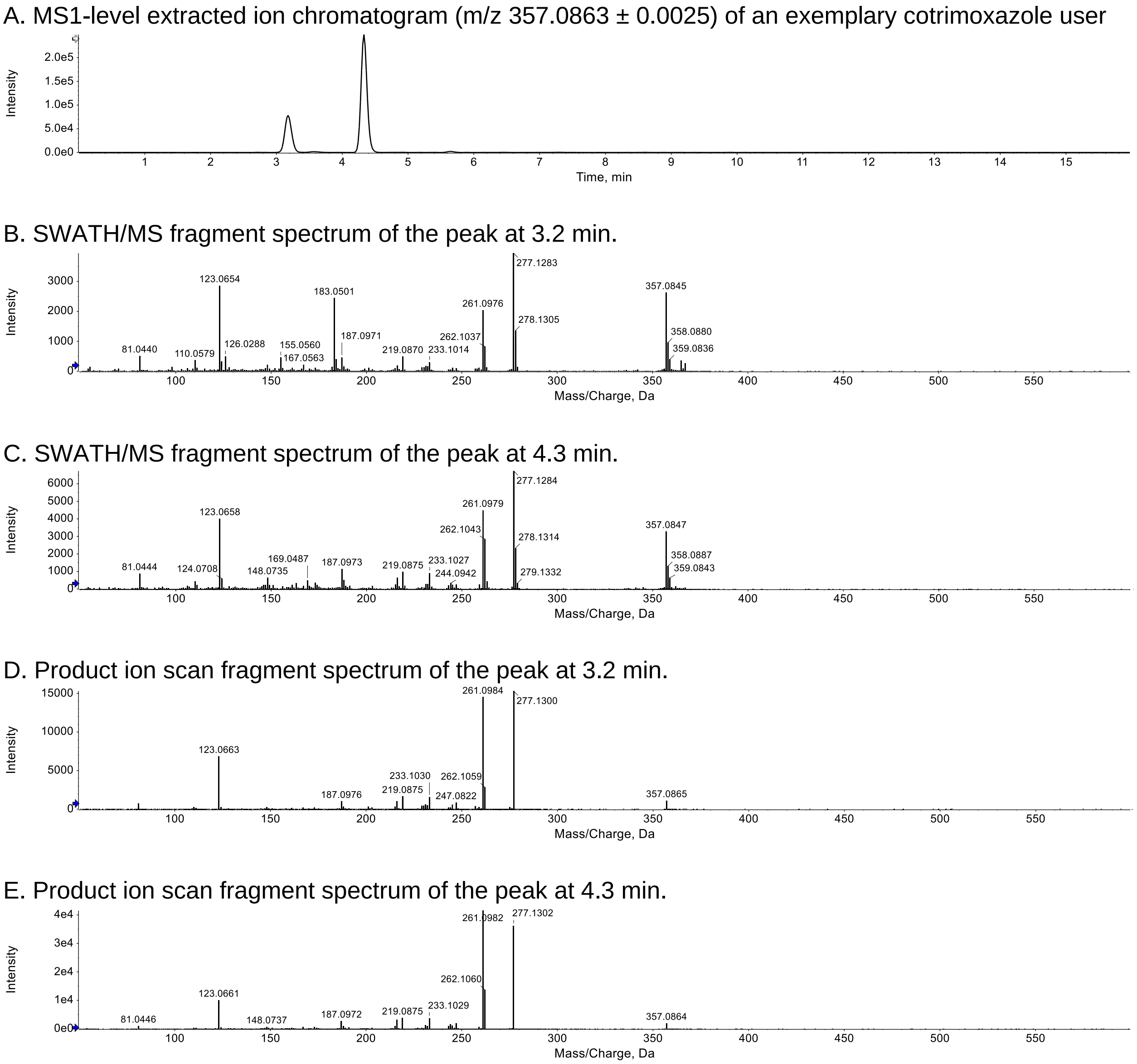

Figure S13. (**A**) MS1-level extracted ion chromatogram, (**B-C**) SWATH/MS fragment spectra, and (**D-E**) product ion scan fragment spectra of two demethylated and sulfated versions of trimethoprim (-14, +80 Da) observed in urine of a human cotrimoxazole user. The substances featured in this figure (possibly being 3-desmethyl trimethoprim sulfate, CID 54390480, and another demethylated and sulfated version of trimethoprim, for which no putative InChI identifier is provided due to the uncertain positions of the removed methyl and added sulfate groups) reflect 'level 3' identifications in terms of the classification proposed by the Metabolomics Standards Initiative (Sumner LW, Amberg A, Barrett D, *et al*. Proposed minimum reporting standards for chemical analysis Chemical Analysis Working Group (CAWG) Metabolomics Standards Initiative (MSI). Metabolomics 2007; 3: 211–221).

A. MS1-level extracted ion chromatogram (m/z 467.1773 ± 0.0025) of an exemplary cotrimoxazole user

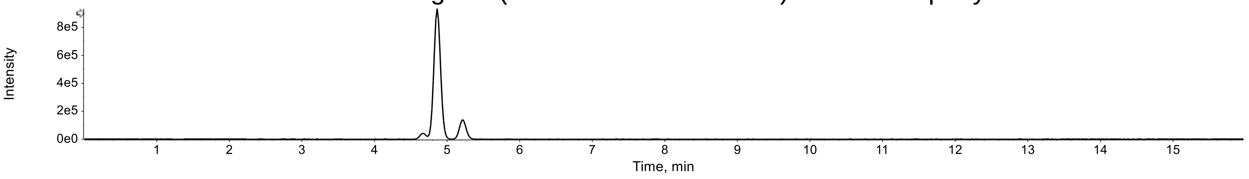

B. SWATH/MS fragment spectrum of the peak at 4.6 min.

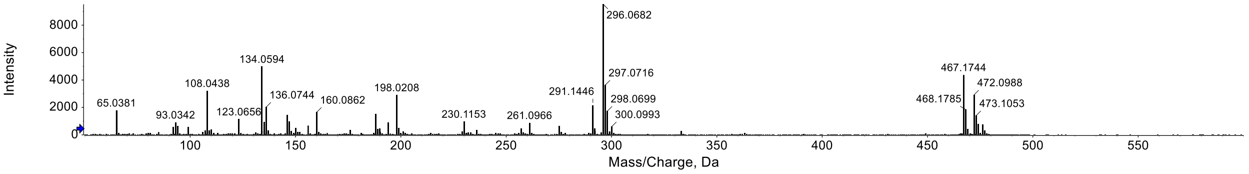

C. SWATH/MS fragment spectrum of the peak at 4.9 min.

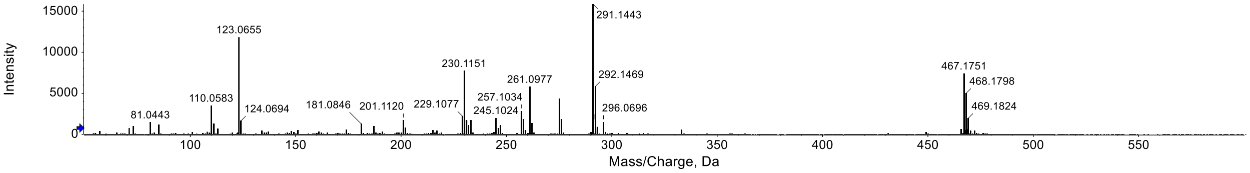

D. SWATH/MS fragment spectrum of the peak at 5.2 min.

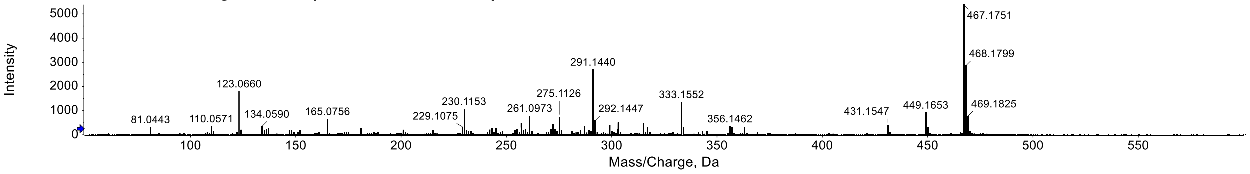

E. Product ion scan fragment spectrum of the peak at 4.6 min.

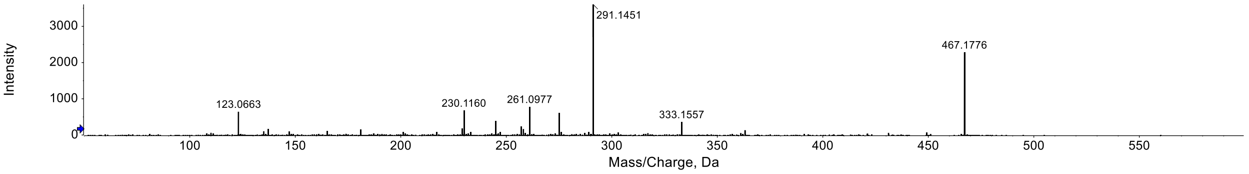

F. Product ion scan fragment spectrum of the peak at 4.9 min.

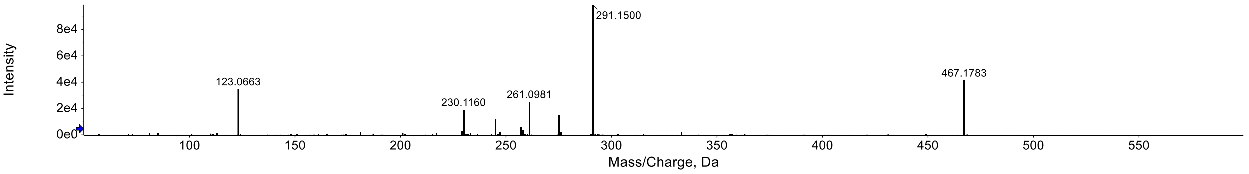

G. Product ion scan fragment spectrum of the peak at 5.2 min.

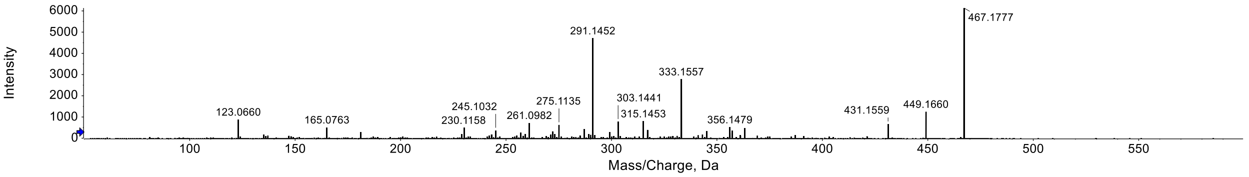

Figure S14. (**A**) MS1-level extracted ion chromatogram, (**B**-**D**) SWATH/MS fragment spectra, and (**E**-**G**) product ion scan fragment spectra of three glucuronidated versions of trimethoprim (+176 Da) observed in urine of a human cotrimoxazole user. The substances featured in this figure (possibly being three different glucuronidated versions of trimethoprim, for which no putative InChI identifiers are provided due to the uncertain positions of the glucuronide moieties) reflect 'level 3' identifications in terms of the classification proposed by the Metabolomics Standards Initiative (Sumner LW, Amberg A, Barrett D, *et al*. Proposed minimum reporting standards for chemical analysis Chemical Analysis Working Group (CAWG) Metabolomics Standards Initiative (MSI). Metabolomics 2007; 3: 211–221).

A. MS1-level extracted ion chromatogram (m/z 483.1722 ± 0.0025) of an exemplary cotrimoxazole user

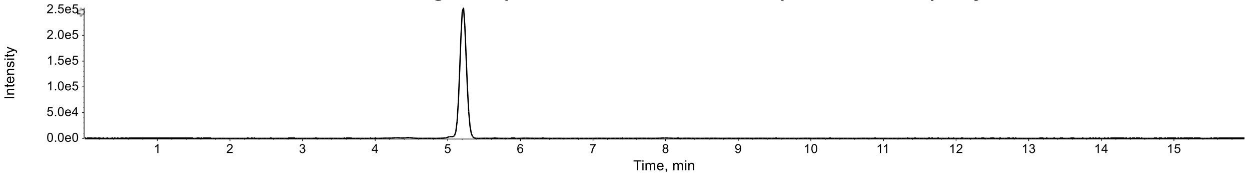

B. SWATH/MS fragment spectrum of the peak at 5.2 min.

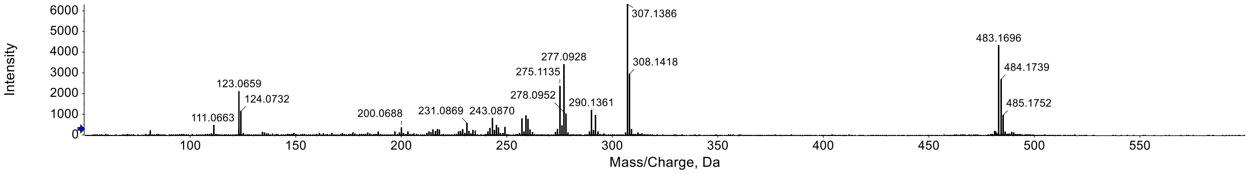

C. Product ion scan fragment spectrum of the peak at 5.2 min.

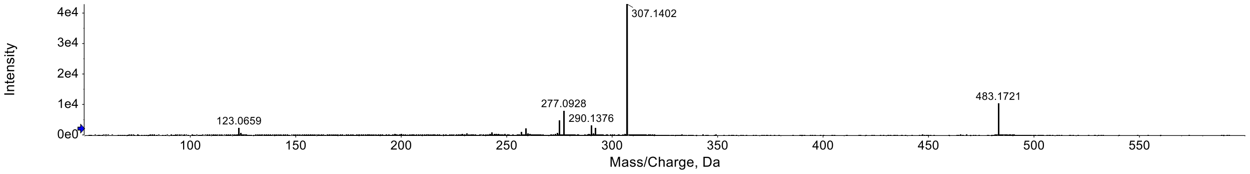

Figure S15. (**A**) MS1-level extracted ion chromatogram, (**B**) SWATH/MS fragment spectrum, and (**C**) product ion scan fragment spectrum of an oxidized and glucuronidated version of trimethoprim (+16, +176 Da) observed in urine of a human cotrimoxazole user. The substance featured in this figure (possibly being alpha-hydroxytrimethoprim glucuronide, CID 169442252) reflects a 'level 3' identification in terms of the classification proposed by the Metabolomics Standards Initiative (Sumner LW, Amberg A, Barrett D, *et al*. Proposed minimum reporting standards for chemical analysis Chemical Analysis Working Group (CAWG) Metabolomics Standards Initiative (MSI). Metabolomics 2007; 3: 211–221).

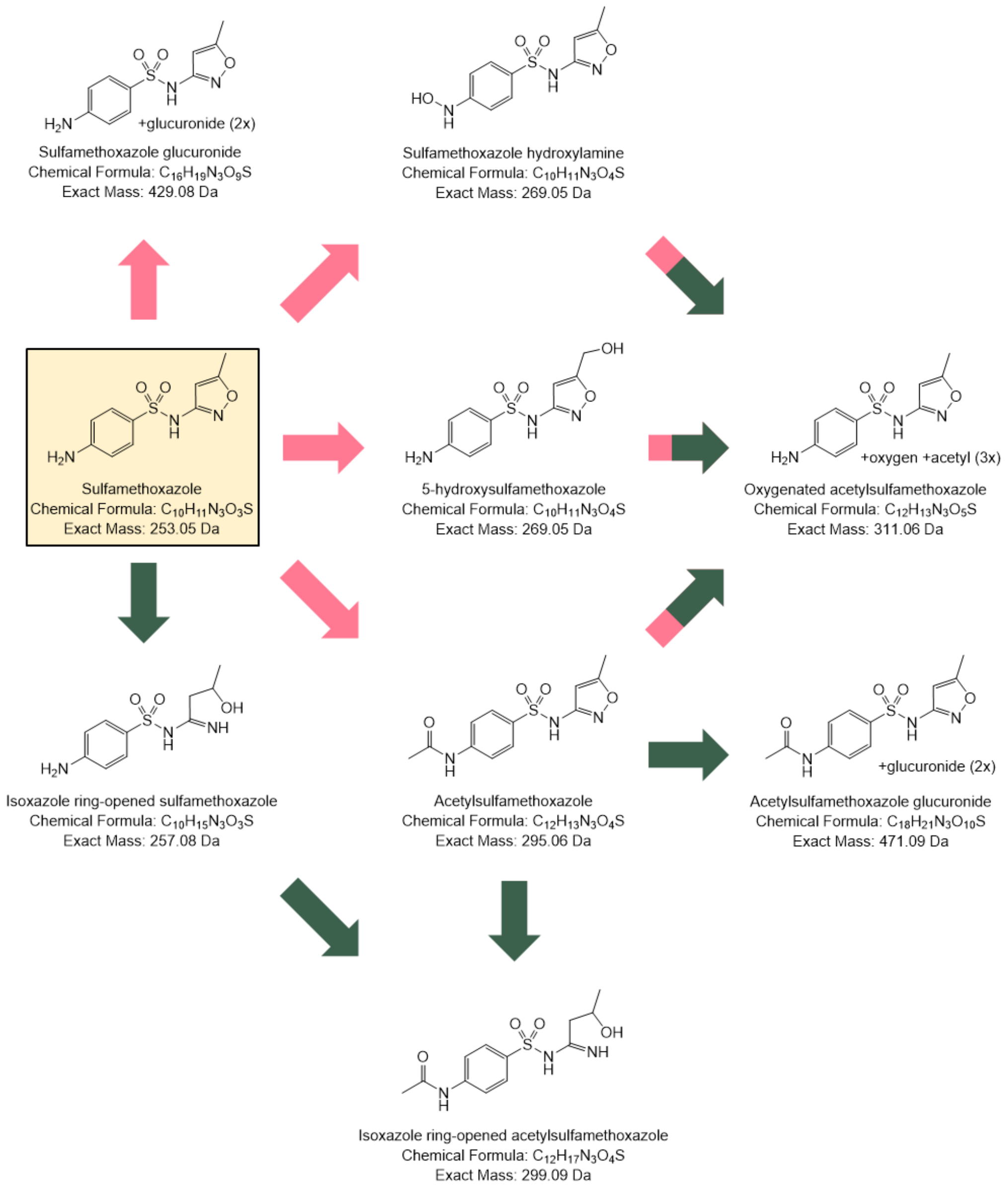

Figure S16. Putative human metabolism of sulfamethoxazole based on this study's findings. The substance depicted in yellow has been confirmed using a chemical reference standard, and noncolored substances represent putatively annotated metabolites. Pink arrows furthermore indicate previously expected conversions, whereas green arrows indicate putative and previously unreported conversions.

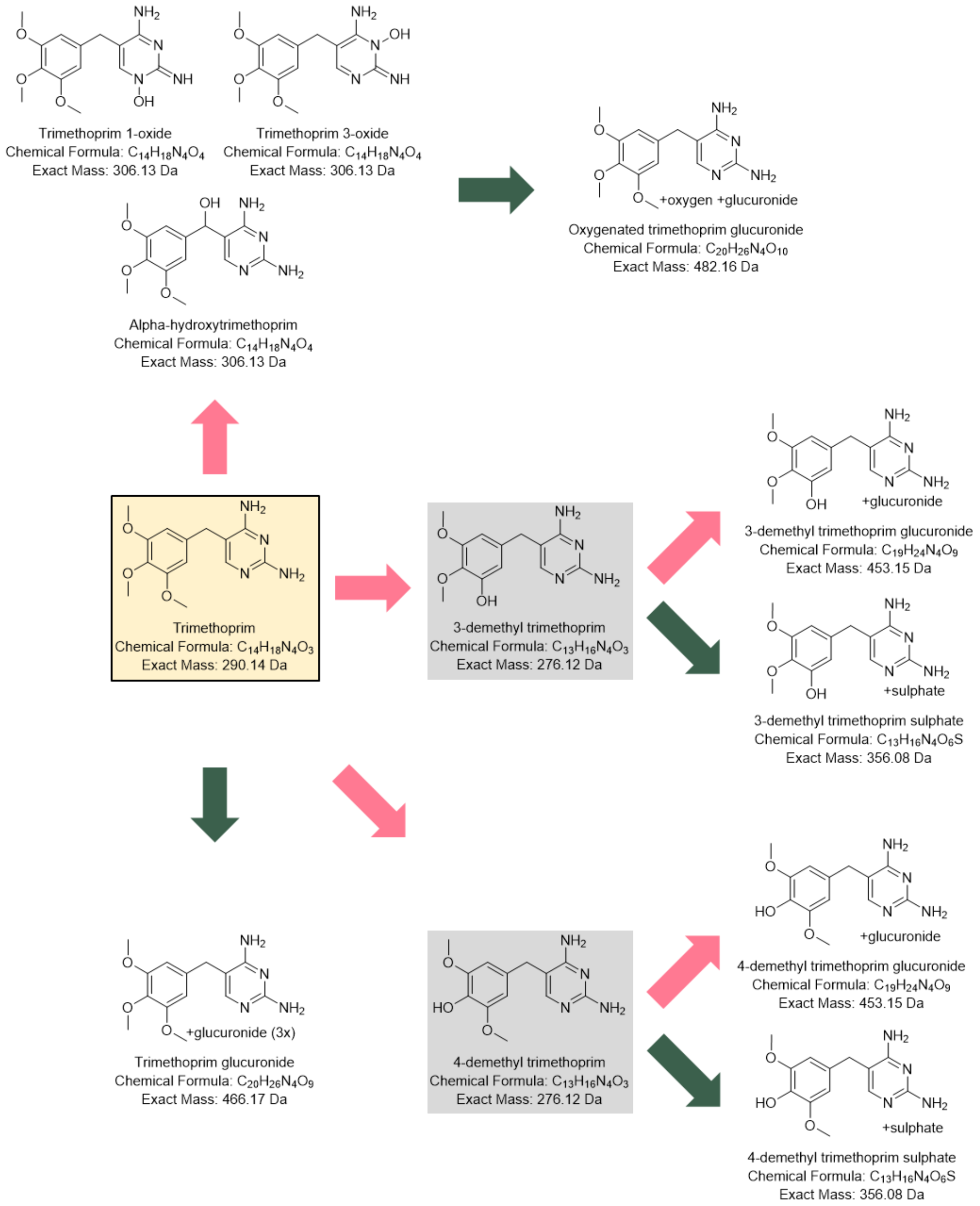

Figure S17. Putative human metabolism of trimethoprim based on this study's findings. The substance depicted in yellow has been confirmed using a chemical reference standard, substances in grey were expected based on previous findings but were not found in this study, and noncolored substances represent putatively annotated metabolites. Pink arrows furthermore indicate previously expected conversions, whereas green arrows indicate putative and previously unreported conversions.

Data S1. Computational details of quantum chemistry simulations.

To investigate the plausibility of the observed biotransformation reactions, quantum chemistry-based simulations were performed using the ‘QCxMS’ method of Koopman and Grimme (J. Am. Soc. Mass Spectrom. 2021; 32: 1735-1751). Initially, plausible structures were generated, guided by the known metabolism of leflunomide, and the isoxazole ring-opening process was hypothesized to yield three possible candidates: the "imine" form, the "keto" form, and the "enol" form (the latter two being tautomers), as illustrated in the figure below. Stability assessments were subsequently conducted by performing automatic protonation of the structures using the open-source Conformer-Rotamer Ensemble Sampling Tool (CREST; J. Chem. Phys., 2024; 160: 114110), followed by Density Functional Theory (DFT) optimization of the obtained $[M\text{-}H]^+$ candidates. The lowest-energy structures were further analyzed and used as input for QCxMS to simulate collision induced dissociation (CID) spectra.

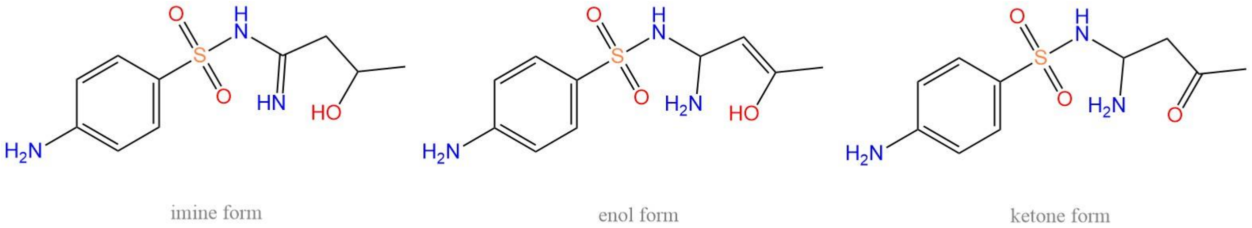

Figure: Proposed structures for isoxazole ring-opened sulfamethoxazole metabolites.

The corresponding stability assessments concluded that the keto and imine forms are essentially equistable, with an energy difference of less than 1 kcal/mol, while the enol form is ~10 kcal/mol less stable. These results were obtained at the ‘PBE/TZPP-D4’ level of theory with Gibbs free energy corrections included.

After evaluating the thermodynamic feasibility of the proposed isoxazole ring-opening, we proceeded to calculate the CID spectra using the QCxMS software. The table below summarizes the cosine similarity scores obtained across a range of simulated CID energies (*i.e.,* 5, 8, 10, 12, 16, 20, 30, 40, 50, 60, 70, 80, 90 eV) and compared them to experimental results. Besides varied CID energies, the default values were used for all other QCxMS parameters.

Table: Cosine similarity scores between experimental CID spectra and simulated spectra for the three proposed isoxazole ring-opened sulfamethoxazole metabolites.

collision energy	enol	imine	ketone	best score
5	0.0163	0.2456	0.2376	imine
8	0.1788	0.2816	0.2591	imine
10	0.0042	0.3517	0.3234	imine
12	0.117	0.4355	0.3934	imine
16	0.1444	0.6322	0.5829	imine
20	0.366	0.7859	0.6712	imine
30	0.751	0.7574	0.537	imine
40	0.6261	0.6073	0.5858	enol
50	0.578	0.5738	0.5167	enol
60	0.5201	0.5203	0.5070	imine
70	0.4872	0.5088	0.4734	imine
80	0.469	0.4846	0.4565	imine

90	0.4632	0.4562	0.4405	enol
highest score	0.751	0.7859	0.6712	
lowest score	0.0042	0.3517	0.3234	
average score	0.4115	0.5558	0.4989	

While exact one-to-one correspondence is challenging, the imine structure consistently exhibited the highest similarity scores. On average, the imine form demonstrated the best overall match, with the highest cosine similarity scores in 10 out of 13 energy levels. In the remaining cases, the enol form, which is presumed to have negligible presence in solution, showed the highest match. Notably, the experimental peak at m/z ~103.08 posed challenges in reproduction through modeling and was only observed with the imine form. Altogether, the results strongly support the imine structure as the most reasonable candidate for the observed sulfamethoxazole metabolite.
